# Supplementary material for: Dutch population structure across space, time and GWAS design
Source: Nat Commun. 2020 Sep 11;11:4556. doi: 10.1038/s41467-020-18418-4 (PMC7486932; doi:10.1038/s41467-020-18418-4)
Supplement: Supplementary file 1 — Supplementary Information [file 41467_2020_18418_MOESM1_ESM.pdf]

Dutch population structure across space, time and GWAS design

Byrne *et al*.

Supplementary material

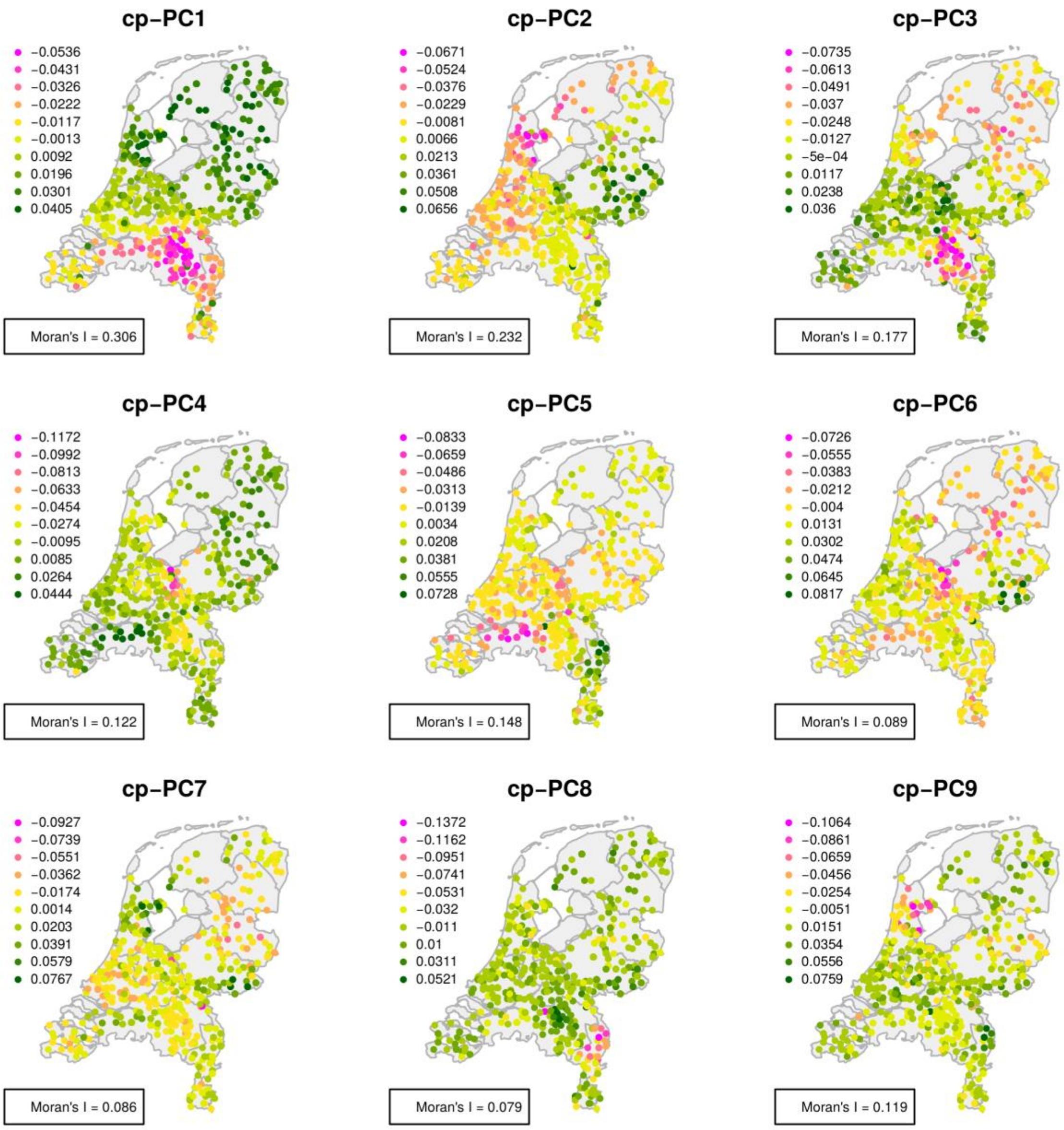

Supplementary Fig. 1 ChromoPainter-PCs relationship to geography. Points on maps are coloured by the average PC value per town of sampling. Each displayed PC shows a significant relationship with geography as tested by Moran's I ($p<0.0001$ for all PCs). PCs have been split into 10 bins for visualisation purposes as in previous works[1]. Map boundary data from the Database of Global Administrative Areas (GADM; https://gadm.org).

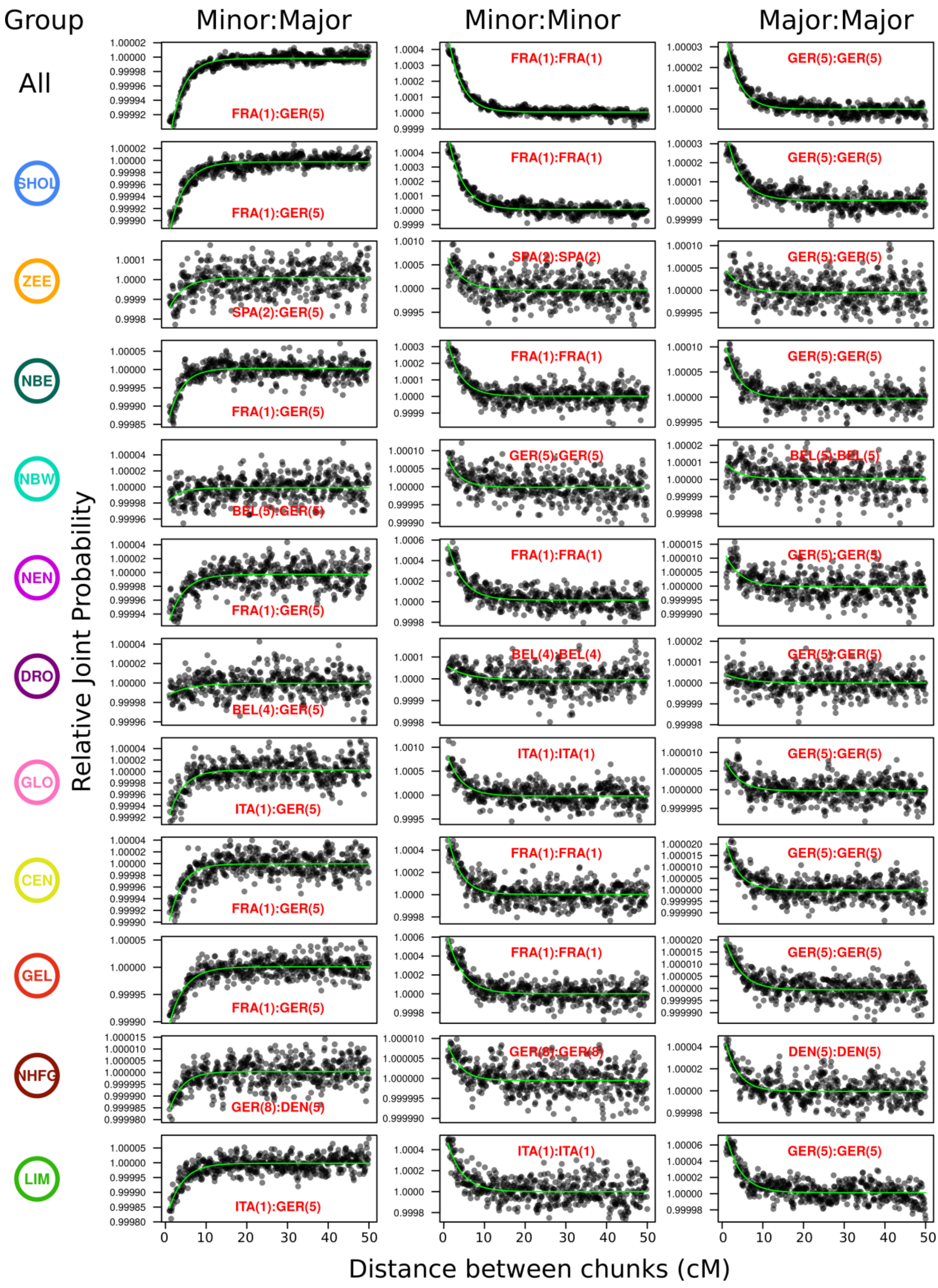

Supplementary Fig. 2 GLOBETROTTER coancestry curves. Example coancestry curves from donors representative of minor and major mixing sources in the GLOBETROTTER analysis of the 12 Dutch groups in Table 1. Each row displays 3 curves for a given Dutch group as labelled on the left (following Fig. 1 and Table 1). The y-axis represents the probability of copying from the two mixing sources (labelled in red eg FRA:GER) at sites separated by a given distance (x-axis; cM). The scaled probabilities are plotted in black, and the GLOBETROTTER fitted curve is in green. Where the fitted slope is positive the mixing sources come from different admixing groups; a negative curve indicates mixing sources come from the same admixing groups.

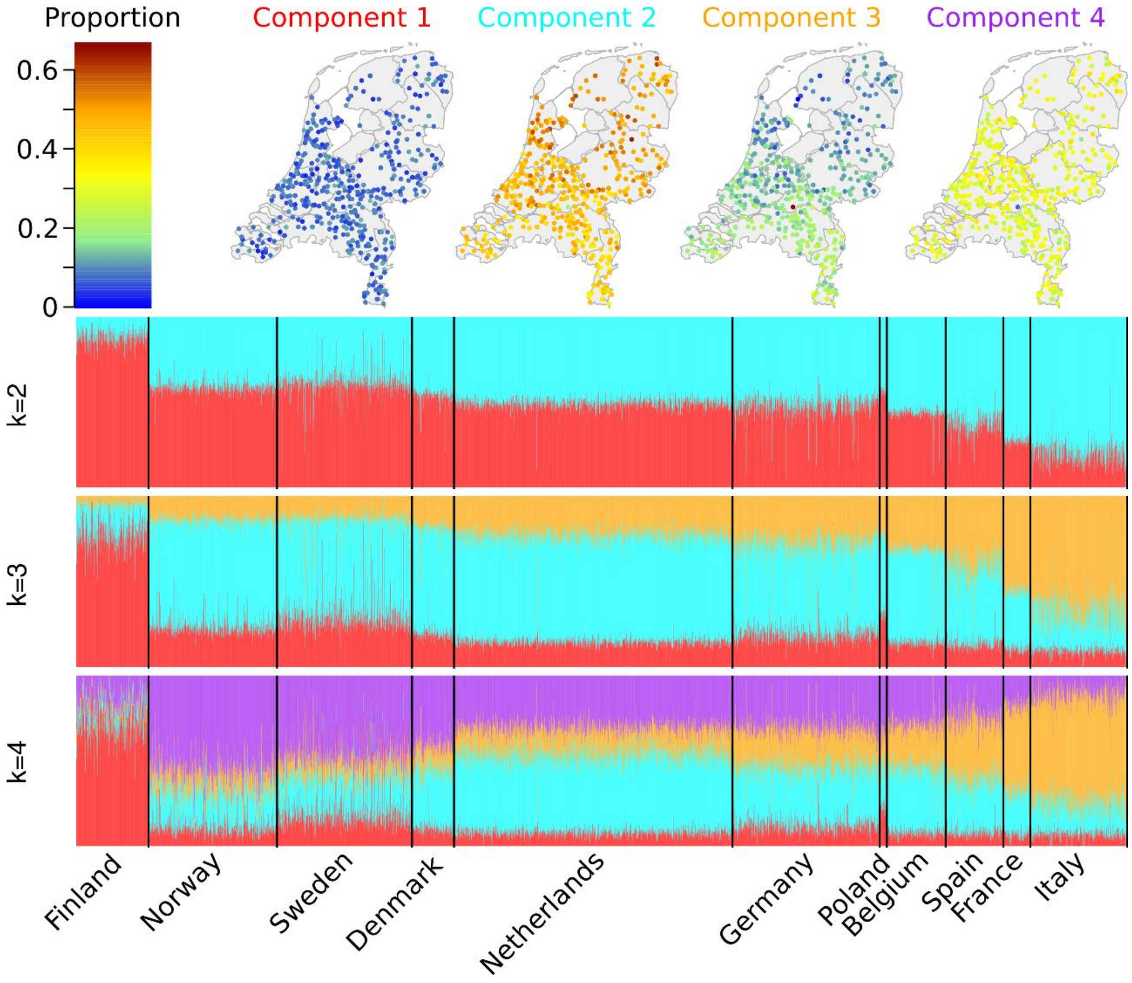

Supplementary Fig. 3 ADMIXTURE modelling for Dutch and European samples. Maps depict the regional breakdown of ADMIXTURE components for k=4 split. Dutch samples have a high value for admixture component 2, which is next highest in Germany and Belgium. Components 2 and 3 show opposing north-south gradients in the Netherlands, with component 2 highest in the north and component 3 highest in the south. Component 3 is best represented in southern European countries such as Italy. Map boundary data from the Database of Global Administrative Areas (GADM; https://gadm.org).

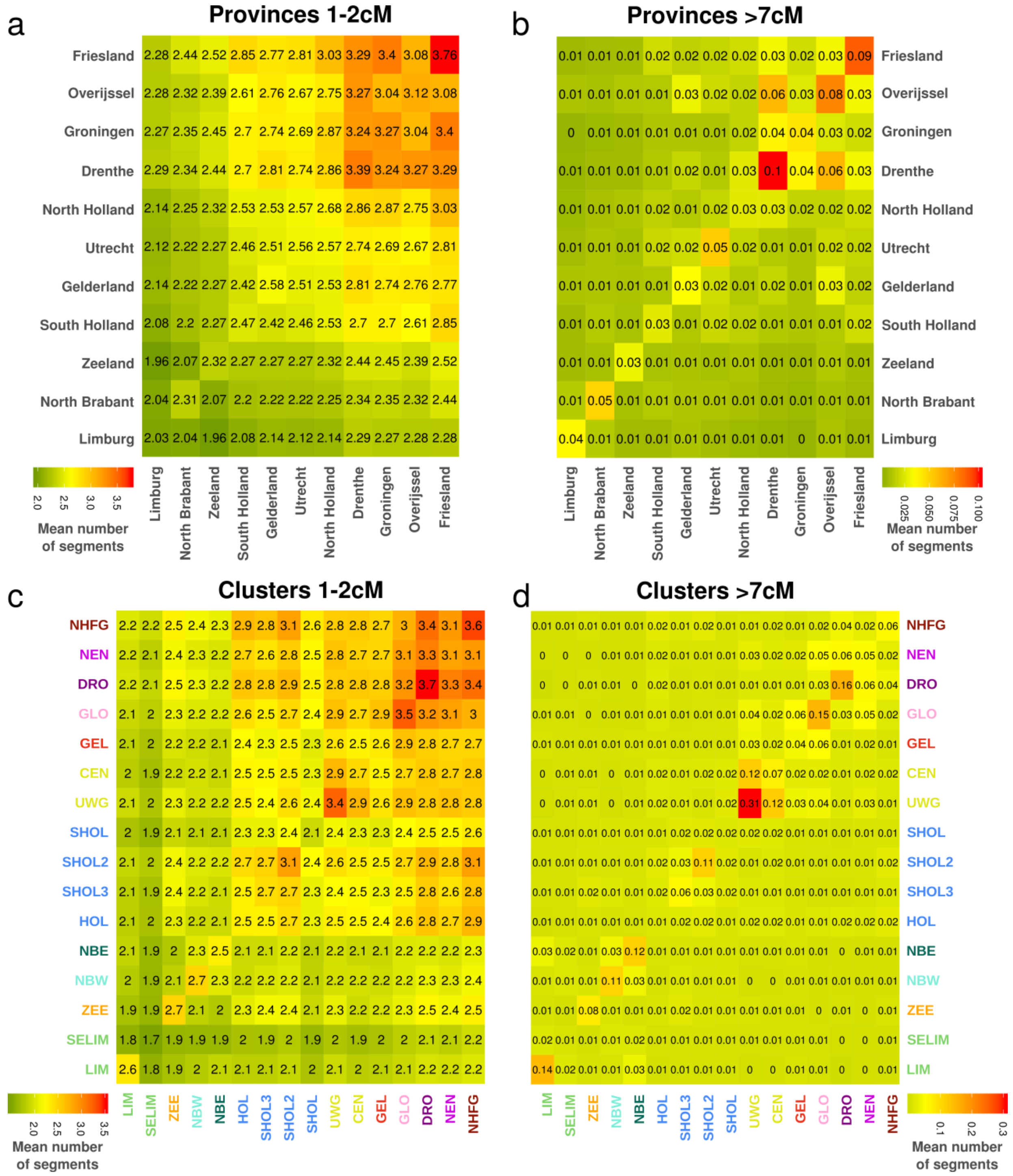

Supplementary Fig. 4 Old and recent IBD sharing per province and per cluster. Average sharing of IBD segments between provinces and clusters respectively is described for old (short) segments (1-2 cM) in (**a**) provinces and (**c**) clusters and for recent (long) segments (>7cM) in (**b**) provinces and (**d**) clusters. Average sharing of old (short) segments is enriched in northern provinces and clusters (a and c). Average sharing of recent (long) segments is higher on average within clusters than within provinces, indicating haplotypic clustering captures marginally more recent ancestry.

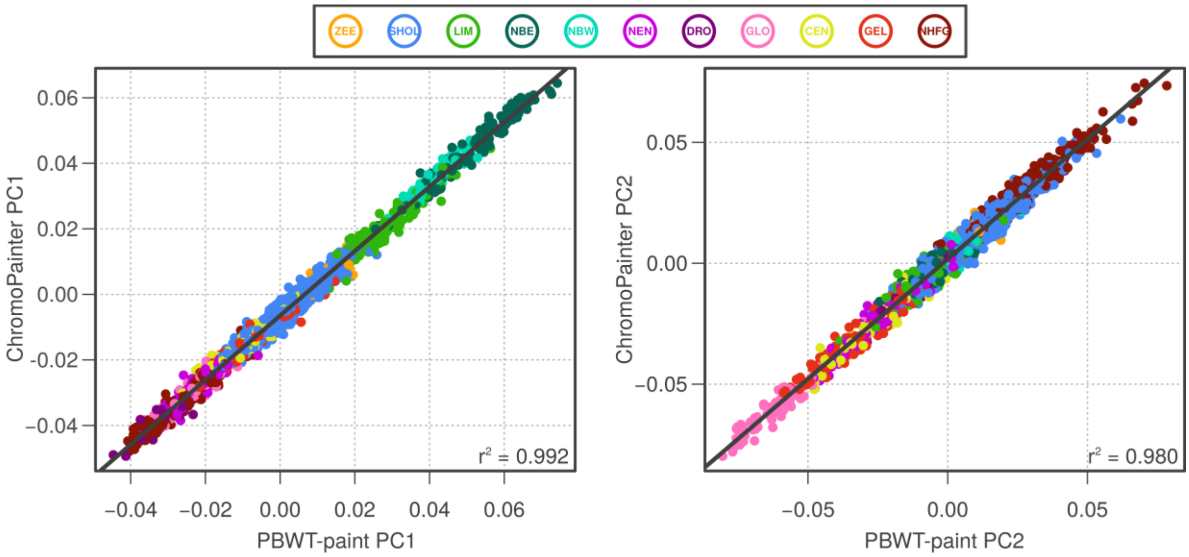

Supplementary Fig. 5 Benchmark of PBWT-paint vs ChromoPainter. Scatterplots comparing the first two principal components (PCs) of the coancestry matrices produced by ChromoPainter and PBWT-paint, showing strong correlation. Points are coloured by cluster groups defined in Fig. 1. For all pairwise comparisons in the two coancestry matrices, Pearson's $\rho = 0.82$ (0.82-0.821; t-statistic = 2332.4 with 2642248 degrees of freedom; $p < 2\times10^{-16}$; exact p-value not reported due to numerical precision limit in R).

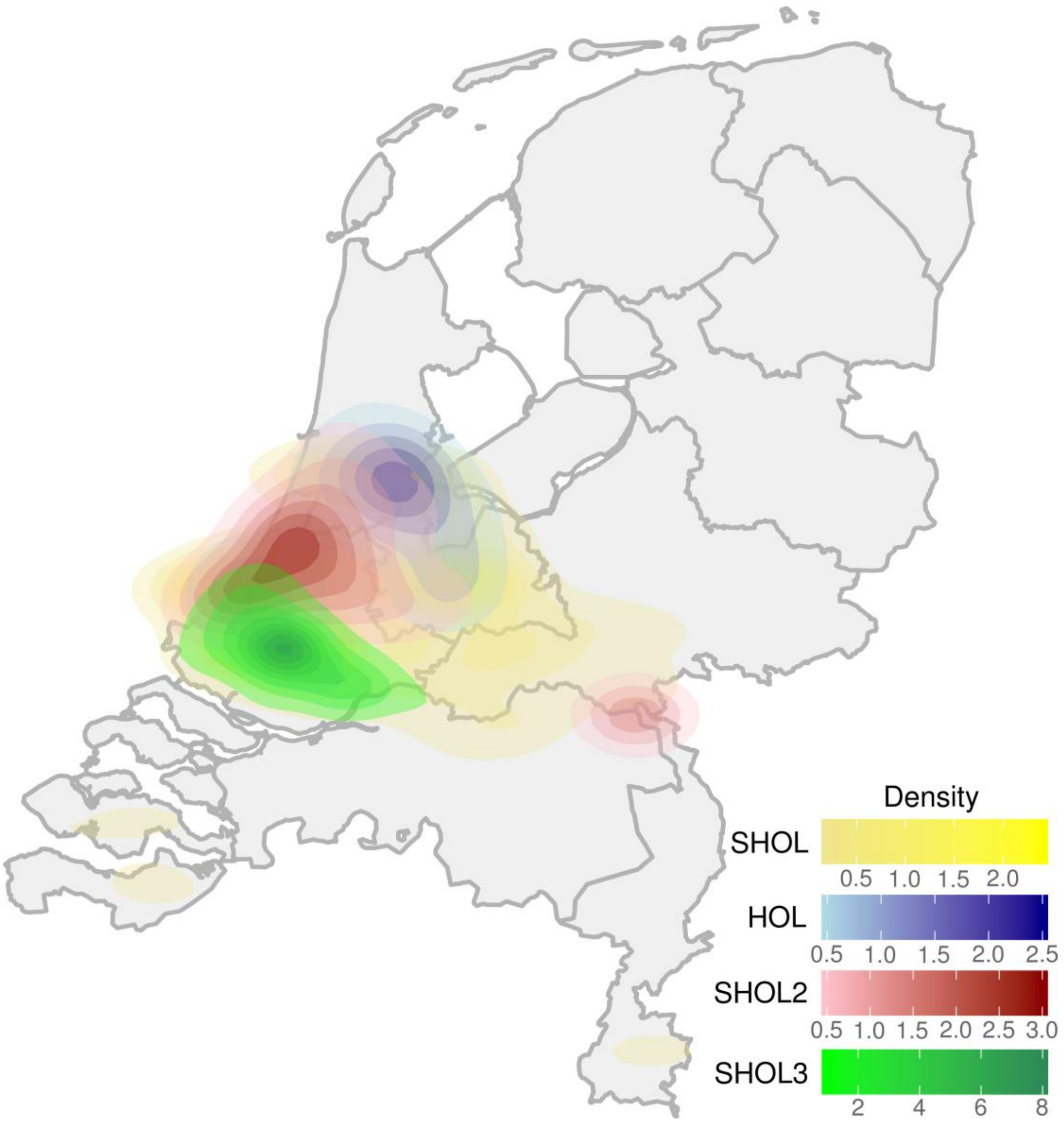

Supplementary Fig. 6 Geographic distribution of South Holland clusters from the SHOL cluster group. 2D kernel density estimates are shown for the geographic spread of samples from clusters SHOL (yellow), HOL (blue), SHOL2 (red), and SHOL3 (green) which form the SHOL cluster group in Fig. 1. Kernel density estimates were calculated using the stat_density2d function in ggplot2 (R version 3.2.3) with default settings. >80% of samples are contained within plotted polygons for each cluster. Notably, although overlapping, three of the four clusters show quite distinct geographic ranges. Map boundary data from the Database of Global Administrative Areas (GADM; https://gadm.org).

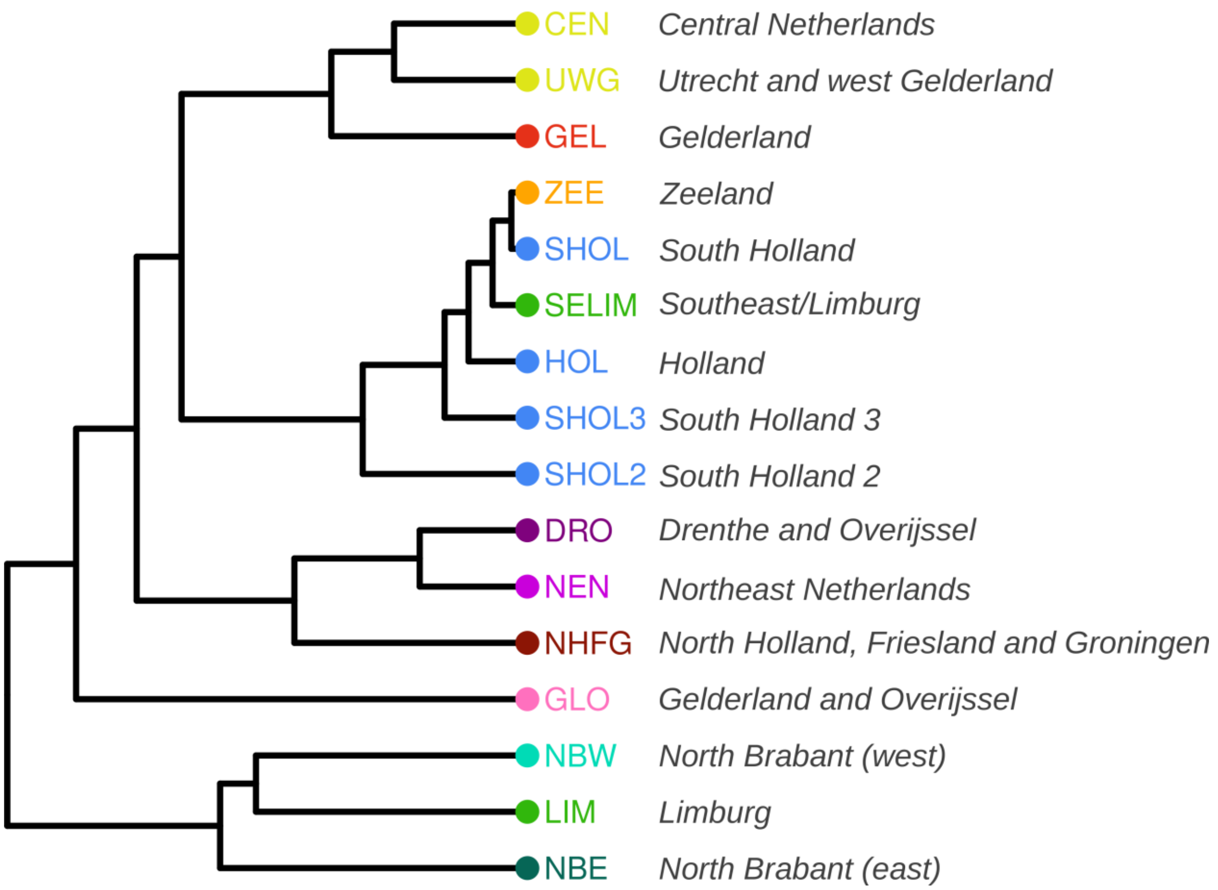

Supplementary Fig. 7 Total variation distance (TVD) tree for k=16 split in the Netherlands. TVD tree analysis provides an alternative view of the relationship between fineSTRUCTURE clusters based on their pairwise TVD scores (Fig. 1a) which should be robust to differences in cluster size. Notably this tree prioritises the split between NBE/NBW/LIM with the rest of the Netherlands, which may reflect the geographic boundary seen in Fig. 5. This tree is based on mean sharing between clusters and may thus miss subtle nuanced relationships where within cluster variation in sharing is non-zero. Clusters are coloured and labelled according to scheme in Fig. 1.

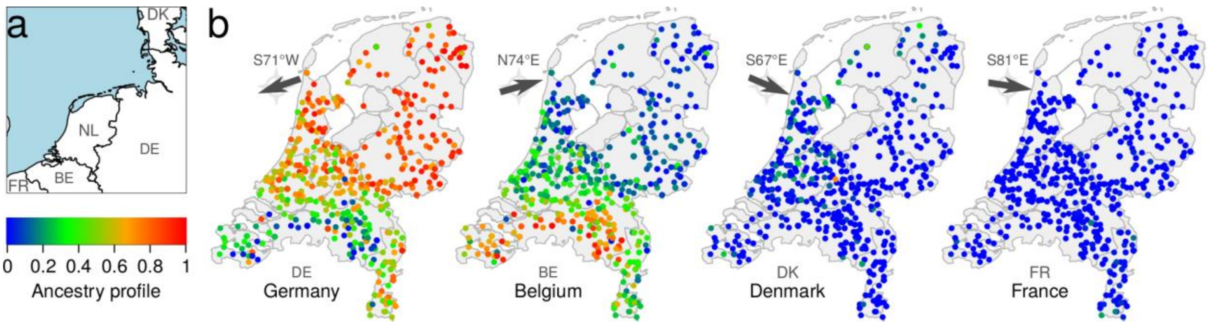

Supplementary Fig. 8 SOURCEFIND ancestry gradients. (**a**) The Netherlands and its geographical relationship to neighbouring lands. (**b**) German, Belgian, Danish and French haplotypic ancestry profiles estimated using the alternative method SOURCEFIND for 1,422 Dutch individuals. Arrows indicate the predominant directions along which the ancestry gradients are arranged across the Netherlands. Major ancestry sources from the NNLS method (Fig. 2) are strongly correlated with these estimates ($\boldsymbol{r^2_{DE} = 0.92}$; $\boldsymbol{r^2_{BE} = 0.97}$; $\boldsymbol{r^2_{DK} = 0.71}$). Map boundary data from the Database of Global Administrative Areas (GADM; https://gadm.org) and Natural Earth.

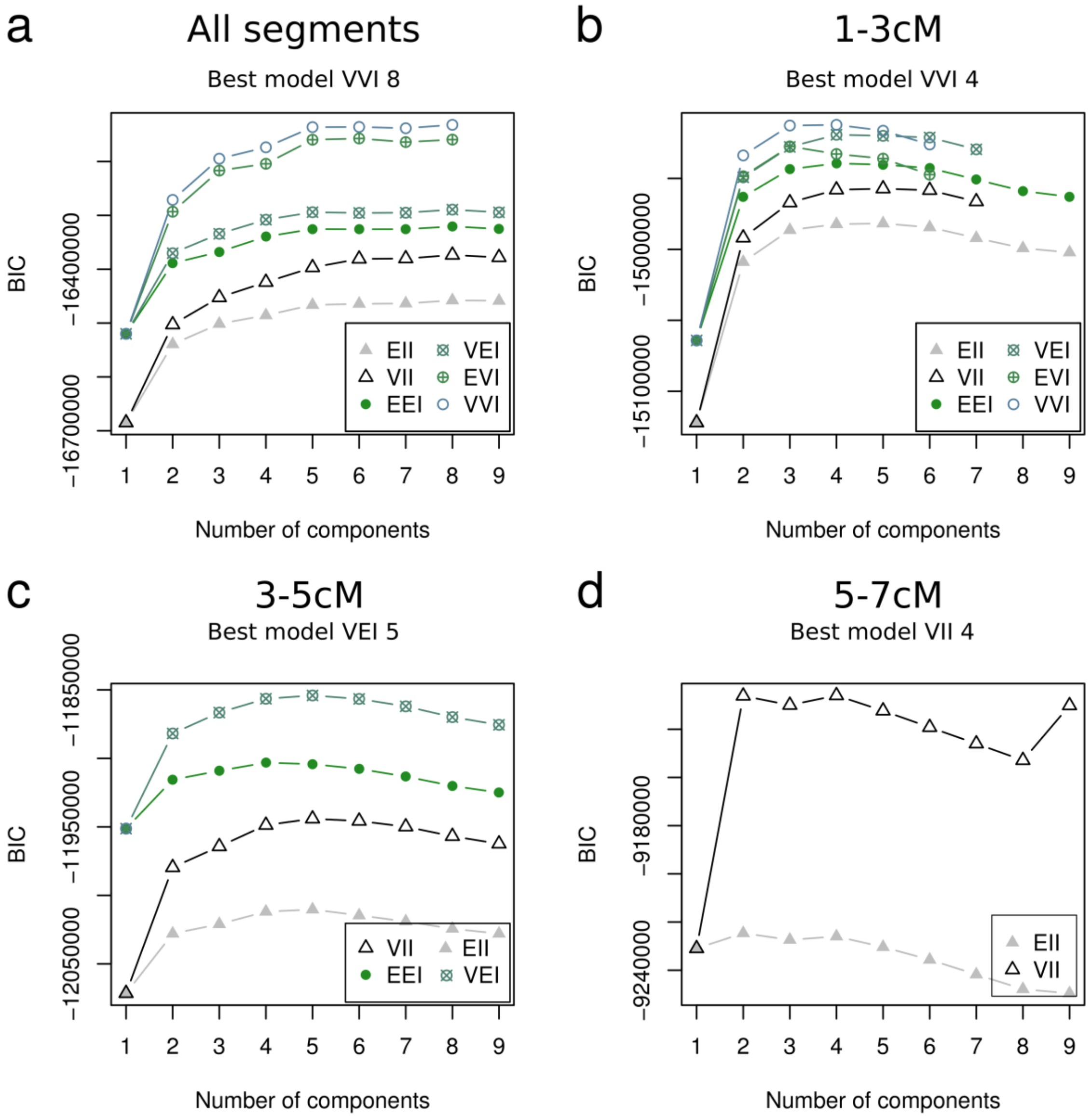

Supplementary Fig. 9 Bayesian information criterion (BIC) versus number of clusters for model choice in mclust. BIC values for mclust models are shown with a range of geometric models (e.g. EII - spherical shape with equal shape and volume[2]) versus number of components fit for IBD sharing datasets in Fig. 3. Models are fit to IBD sharing matrices between Dutch individuals including (**a**) segments of all lengths, (**b**) segments of lengths 1-3cM, (**c**) segments of lengths 3-5cM and (**d**) segments of lengths 5-7cM. The model with the maximum BIC was chosen for clustering for each IBD bin. Note that missing values here represent instances where models could not be estimated due to occurrence of a singularity in the covariance matrix often resulting from one or more components being fit to a single point. Under these conditions model likelihood trends to infinity and is uninterpretable. Legend abbreviations represent models with different geometric characteristics described in the mclust paper[2].

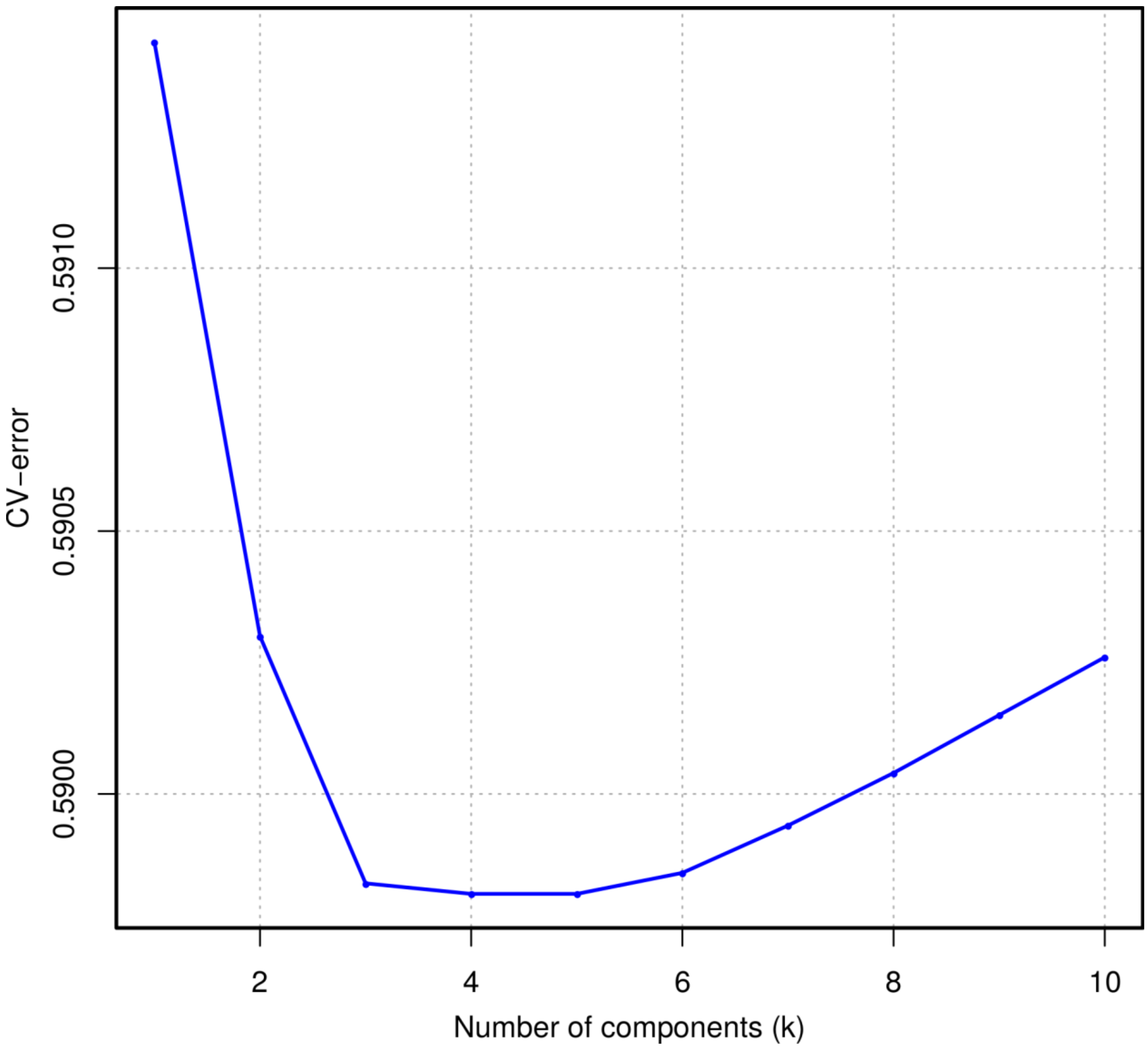

Supplementary Fig. 10 ADMIXTURE CV-error plot. Displays the cross-validation (CV) error for ADMIXTURE run with 1-10 components. Models with 4 and 5 components are tied for lowest CV error, suggesting choice of either is suitable.

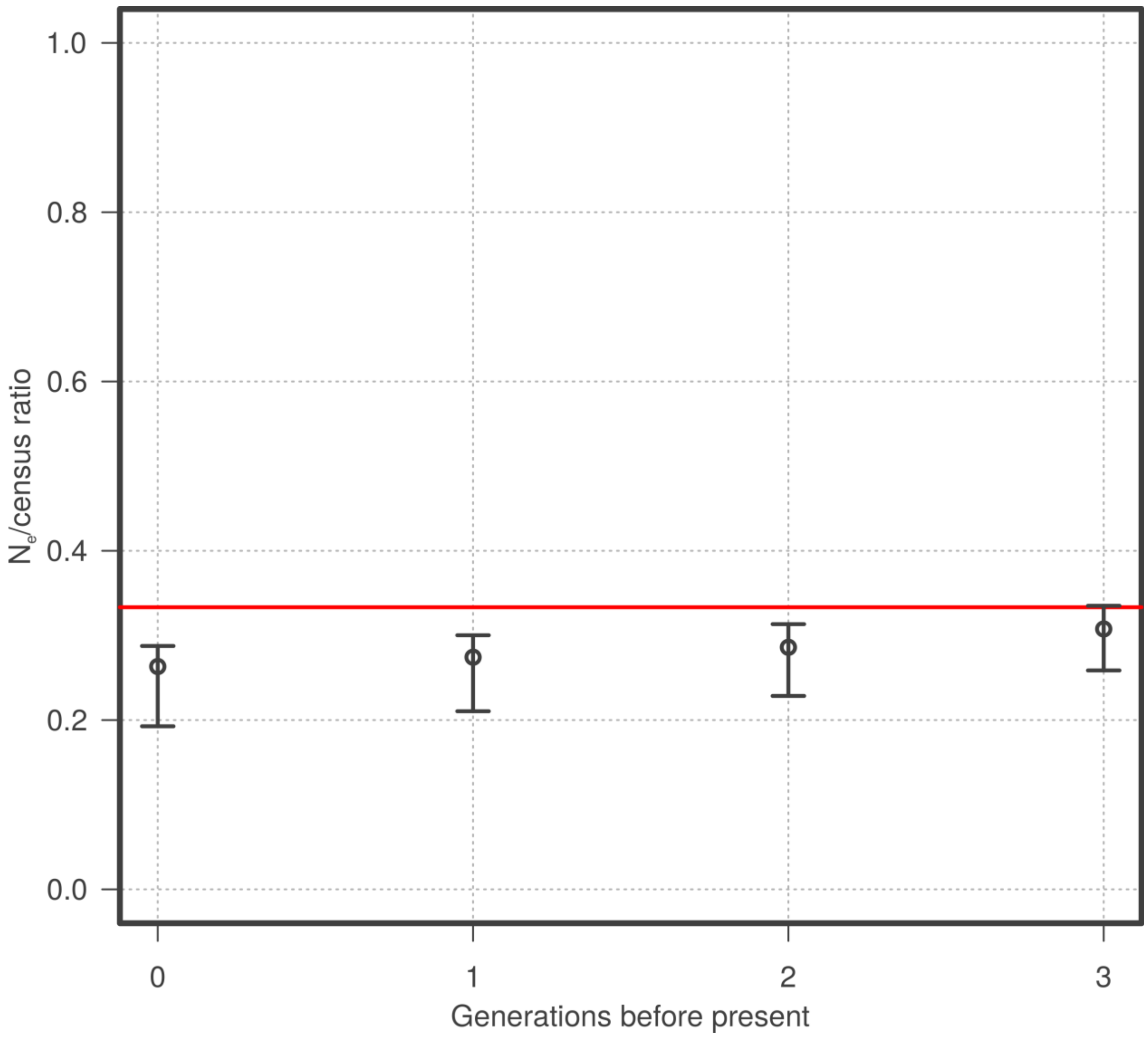

Supplementary Fig. 11 Ratio of estimated Ne/Census is stable over the past 3 generations. The red line at 0.33 corresponds to the expected ratio of N_e to census if lifespan is 3 times the generation time. Point estimates represent the ratio of estimated N_e to census value for a given generation in the full Netherlands dataset, while error bars represent 95% confidence interval for this value calculated using 80 bootstrap resamples in IBDNe (note this is not necessarily symmetric on the point estimate).

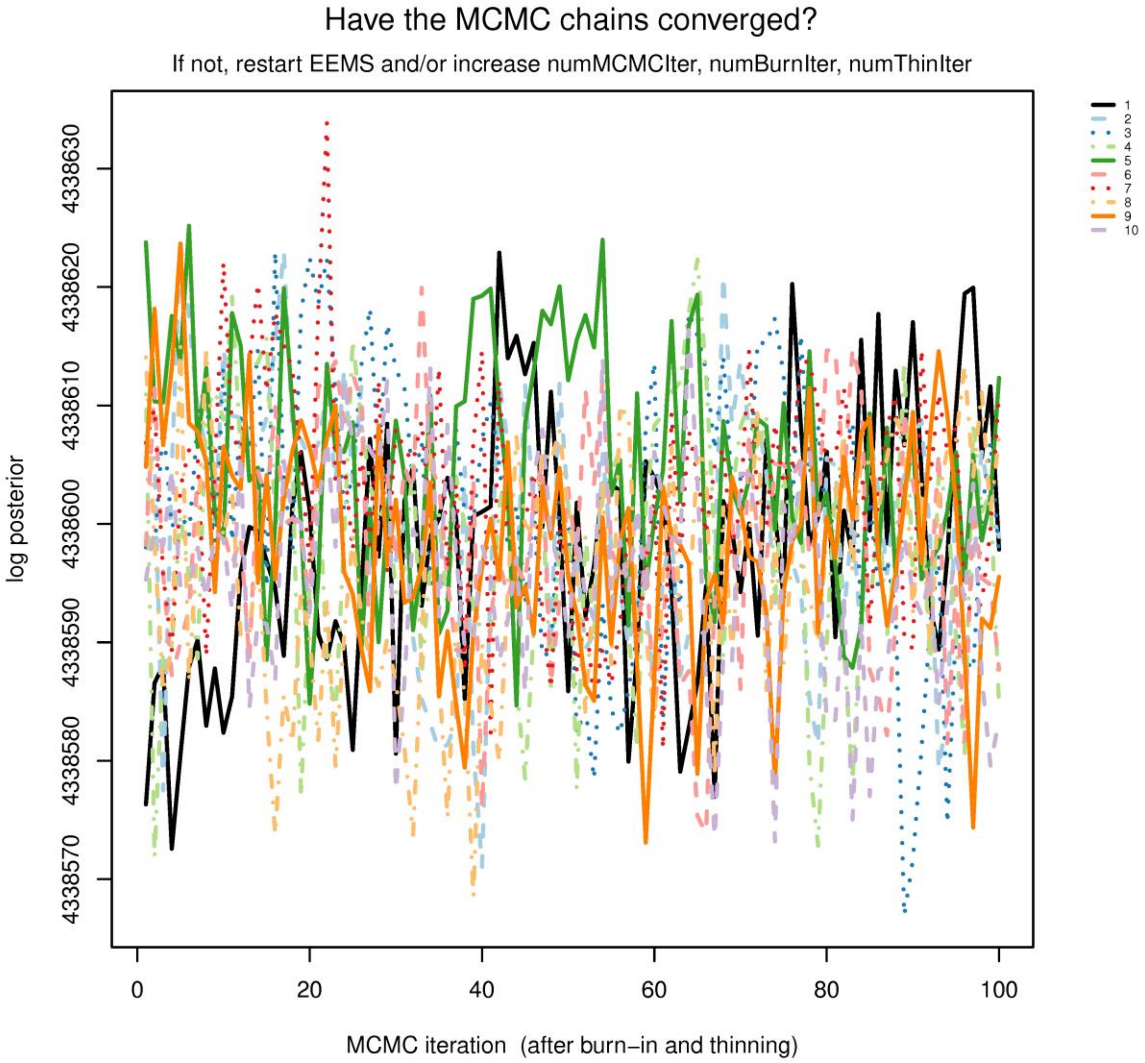

Supplementary Fig. 12 Convergence of MCMC chains for EEMS run in The Netherlands. 10 independently seeded MCMC chains reach approximate convergence.

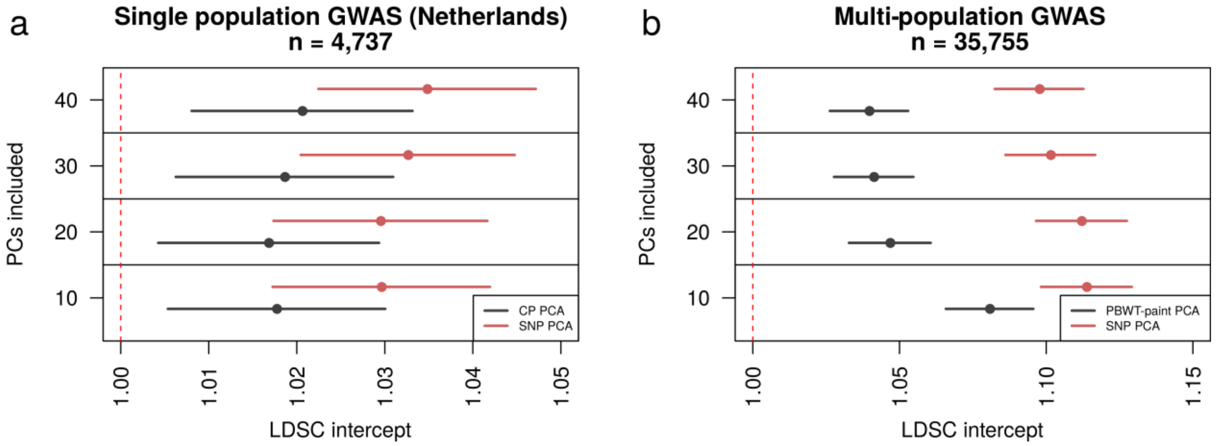

Supplementary Fig. 13 LDSC intercepts from ALS GWAS using haplotype PCs vs SNP PCs. Displays LDSC intercepts (points) and 95% confidence intervals (whiskers) for (**a**) a single population GWAS and (**b**) a multi-population GWAS of ALS, fitting PCs calculated from haplotype sharing matrices (black) and SNPs (red) as covariates. Using PCs from haplotype sharing matrices reduces the LDSC intercept relative to using SNP PCs, suggesting haplotype sharing matrices correct for confounding not captured by SNP PCA. Error bars represent 95% confidence interval centred on the LDSC intercepts. Abbreviations: CP PCA, ChromoPainter PCA; LDSC, LD score regression; PBWT-paint, positional Burrows-Wheeler transform-paint.

Supplementary Table 1 Mean pairwise F_{ST} ($\times 10^{-3}$) for Dutch clusters and European groups from Sawcer *et al*.[3]

	Finland	Sweden	Norway	Germany	Italy	Denmark	Belgium	Spain	Poland	France	ZEE	SHOL	HOL	SHOL2	SHOL3	LIM	SELIM	NBE	NBW	NEN	DRO	GLO	UWG	CEN	GEL	NHFG
**Finland**	0.0	-	-	-	-	-	-	-	-	-	-	-	-	-	-	-	-	-	-	-	-	-	-	-	-	-
**Sweden**	3.9	0.0	-	-	-	-	-	-	-	-	-	-	-	-	-	-	-	-	-	-	-	-	-	-	-	-
**Norway**	5.1	0.4	0.0	-	-	-	-	-	-	-	-	-	-	-	-	-	-	-	-	-	-	-	-	-	-	-
**Germany**	5.9	0.7	0.9	0.0	-	-	-	-	-	-	-	-	-	-	-	-	-	-	-	-	-	-	-	-	-	-
**Italy**	11.1	4.9	5.0	2.8	0.0	-	-	-	-	-	-	-	-	-	-	-	-	-	-	-	-	-	-	-	-	-
**Denmark**	5.6	0.4	0.4	0.4	4.1	0.0	-	-	-	-	-	-	-	-	-	-	-	-	-	-	-	-	-	-	-	-
**Belgium**	6.7	1.1	1.1	0.3	2.3	0.5	0.0	-	-	-	-	-	-	-	-	-	-	-	-	-	-	-	-	-	-	-
**Spain**	10.0	3.8	3.7	2.1	1.2	3.0	1.5	0.0	-	-	-	-	-	-	-	-	-	-	-	-	-	-	-	-	-	-
**Poland**	5.8	1.8	2.6	1.3	5.4	2.1	2.4	4.6	0.0	-	-	-	-	-	-	-	-	-	-	-	-	-	-	-	-	-
**France**	7.6	1.8	1.8	0.7	1.6	1.2	0.4	0.7	2.8	0.0	-	-	-	-	-	-	-	-	-	-	-	-	-	-	-	-
**ZEE**	6.8	1.0	1.0	0.5	3.4	0.5	0.3	2.4	2.7	0.9	0.0	-	-	-	-	-	-	-	-	-	-	-	-	-	-	-
**SHOL**	6.4	0.8	0.8	0.3	3.2	0.3	0.2	2.3	2.4	0.8	0.2	0.0	-	-	-	-	-	-	-	-	-	-	-	-	-	-
**HOL**	6.4	0.8	0.8	0.4	3.7	0.3	0.4	2.7	2.5	1.0	0.2	0.0	0.0	-	-	-	-	-	-	-	-	-	-	-	-	-
**SHOL2**	6.7	1.0	1.0	0.7	4.2	0.5	0.7	3.1	2.9	1.4	0.5	0.3	0.2	0.0	-	-	-	-	-	-	-	-	-	-	-	-
**SHOL3**	6.5	0.9	0.9	0.5	3.7	0.4	0.4	2.7	2.6	1.1	0.2	0.1	0.1	0.2	0.0	-	-	-	-	-	-	-	-	-	-	-
**LIM**	7.1	1.4	1.4	0.7	3.0	0.8	0.5	2.2	2.8	0.9	0.6	0.5	0.6	0.8	0.7	0.0	-	-	-	-	-	-	-	-	-	-
**SELIM**	6.5	0.9	0.9	0.2	2.5	0.4	0.1	1.8	2.2	0.5	0.3	0.1	0.2	0.6	0.3	0.3	0.0	-	-	-	-	-	-	-	-	-
**NBE**	6.8	1.2	1.2	0.6	3.3	0.7	0.4	2.4	2.7	1.0	0.6	0.4	0.5	0.8	0.6	0.6	0.3	0.0	-	-	-	-	-	-	-	-
**NBW**	6.9	1.3	1.3	0.7	3.3	0.8	0.4	2.4	2.9	1.1	0.6	0.5	0.5	0.9	0.5	0.8	0.4	0.5	0.0	-	-	-	-	-	-	-
**NEN**	6.5	0.9	0.9	0.6	4.3	0.4	0.6	3.2	2.8	1.4	0.5	0.2	0.1	0.4	0.3	0.8	0.5	0.7	0.8	0.0	-	-	-	-	-	-
**DRO**	6.9	1.1	1.1	0.9	4.7	0.6	0.9	3.6	3.0	1.8	0.6	0.5	0.3	0.6	0.4	1.0	0.7	1.1	1.1	0.1	0.0	-	-	-	-	-
**GLO**	6.8	1.2	1.2	0.9	4.4	0.7	0.9	3.4	3.1	1.6	0.6	0.5	0.5	0.8	0.6	1.1	0.7	1.0	1.0	0.2	0.6	0.0	-	-	-	-
**UWG**	7.0	1.3	1.3	1.0	4.3	0.8	0.9	3.2	3.0	1.6	0.7	0.6	0.6	0.9	0.7	1.0	0.7	1.1	1.0	0.6	0.8	0.8	0.0	-	-	-
**CEN**	6.6	0.9	0.9	0.5	3.7	0.4	0.4	2.7	2.5	1.1	0.3	0.1	0.1	0.3	0.2	0.7	0.3	0.6	0.6	0.2	0.5	0.4	0.0	0.0	-	-
**GEL**	6.4	0.8	0.8	0.4	3.6	0.3	0.3	2.6	2.6	1.0	0.3	0.1	0.1	0.4	0.2	0.6	0.2	0.5	0.5	0.1	0.4	0.1	0.5	0.0	0.0	-
**NHFG**	6.6	1.1	1.0	0.9	4.7	0.6	0.9	3.6	3.1	1.7	0.6	0.4	0.2	0.4	0.3	1.1	0.7	1.0	1.0	0.2	0.3	0.7	0.9	0.4	0.4	0.0

Supplementary note 1

Project MinE ALS GWAS Consortium authors

Wouter van Rheenen[1], Aleksey Shatunov[2], Russell L. McLaughlin[3], Rick A.A. van der Spek[1], Alfredo Iacoangeli[2,4], Kevin P. Kenna[1], Kristel R. van Eijk[1], Nicola Ticozzi[5,6], Boris Rogelj[7,8], Katarina Vrabec[9], Metka Ravnik-Glavač[9,10], Blaž Koritnik[11], Janez Zidar[11], Lea Leonardis[11], Leja Dolenc Grošelj[11], Stéphanie Millecamps[12], François Salachas[12,13,14], Vincent Meininger[15,16] , Mamede de Carvalho[17,18] , Susana Pinto[17], Marta Gromicho[17], Ana Pronto-Laborinho[17], Jesus S. Mora[19], Ricardo Rojas-García[20,21], Meraida Polak[22,23], Siddharthan Chandran[24,25], Shuna Colville[24], Robert Swingler[24], Karen E. Morrison[26], Pamela J. Shaw[27], John Hardy[28], Richard W. Orrell[29], Alan Pittman[28,30], Katie Sidle[29], Pietro Fratta[31], Andrea Malaspina[32,33], Simon Topp[2], Susanne Petri[34], Susanna Abdulla[35], Carsten Drepper[36], Michael Sendtner[36], Thomas Meyer[37], Roel A. Ophoff[38,39], Kim A. Staats[39], Martina Wiedau-Pazos[40], Catherine Lomen-Hoerth[41], Vivianna M. Van Deerlin[42], John Q. Trojanowski[42], Lauren Elman[43], Leo McCluskey[43], A. Nazli Basak[44], Thomas Meitinger[45], Peter Lichtner[45], Milena Blagojevic-Radivojkov[45], Christian R. Andres[46], Gilbert Bensimon[47,48,49], Bernhard Landwehrmeyer[50], Alexis Brice[51,52,53,54,55], Christine A.M. Payan[47,49], Safaa Saker-Delye[56], Alexandra Dürr[57], Nicholas W. Wood[58], Lukas Tittmann[59], Wolfgang Lieb[59], Andre Franke[60], Marcella Rietschel[61], Sven Cichon[62,63,64,65,66], Markus M. Nöthen[62,63], Philippe Amouyel[67], Jean-François Dartigues[68], Andre G. Uitterlinden[69,70], Fernando Rivadeneira[69,70], Karol Estrada[69], Albert Hofman[70,71], Charles Curtis[72,73], Anneke J. van der Kooi[74], Markus Weber[75], Christopher E. Shaw[2], Bradley N. Smith[2], Daisy Sproviero[76], Cristina Cereda[76], Mauro Ceroni[77], Luca Diamanti[77], Roberto Del Bo[78], Stefania Corti[78], Giacomo P. Comi[78], Sandra D'Alfonso[79], Lucia Corrado[79], Cinzia Bertolin[80], Gianni Sorarù[80], Letizia Mazzini[81], Viviana Pensato[82], Cinzia Gellera[82], Cinzia Tiloca[5], Antonia Ratti[5,6], Andrea Calvo[83,84], Cristina Moglia[83,84], Maura Brunetti[83,84], Rosa Capozzo[85], Chiara Zecca[85], Christian Lunetta[86], Silvana Penco[87], Nilo Riva[88], Alessandro Padovani[89], Massimiliano Filosto[90], PARALS registry[91], SLALOM group[91], SLAP registry[91], SLAGEN Consortium[91], NNIPPS Study Group[91], Ian Blair[92], Garth A. Nicholson[92,93], Dominic B. Rowe[92], Roger Pamphlett[94], Matthew C. Kiernan[95], Julian Grosskreutz[96], Otto W. Witte[96], Robert Steinbach[96], Tino Prell[96], Beatrice Stubendorff[96], Ingo Kurth[97,98], Christian A. Hübner[97], P. Nigel Leigh[99], Federico Casale[83], Adriano Chio[83,84], Ettore Beghi[100], Elisabetta Pupillo[100], Rosanna Tortelli[101], Giancarlo Logroscino[102,103], John Powell[2], Albert C. Ludolph[50], Jochen H. Weishaupt[50], Wim Robberecht[104,105,106], Philip Van Damme[104,105,106], Robert H. Brown[107], Jonathan D. Glass[22,23], John E. Landers[107], Orla Hardiman[108,109], Peter M. Andersen[110], Philippe Corcia[46,111,112] , Patrick Vourc'h[46], Vincenzo Silani[5,6], Michael A. van Es[1], R. Jeroen Pasterkamp[113], Cathryn M. Lewis[72,114], Gerome Breen[72,73], Ammar Al-Chalabi[2], Leonard H. van den Berg[1], Jan H. Veldink[1]

1. Department of Neurology, UMC Utrecht Brain Center, University Medical Center Utrecht, Utrecht University, Utrecht, The Netherlands.
2. Maurice Wohl Clinical Neuroscience Institute, King's College London, Department of Basic and Clinical Neuroscience, London, UK.
3. Complex Trait Genomics Laboratory, Smurfit Institute of Genetics, Trinity College Dublin, Dublin, Republic of Ireland.
4. Department of Biostatistics and Health Informatics, Institute of Psychiatry, Psychology and Neuroscience, King's College London, London, UK.

5. Department of Neurology and Laboratory of Neuroscience, IRCCS Istituto Auxologico Italiano, Milano, Italy.
6. Department of Pathophysiology and Tranplantation, ‘Dino Ferrari’ Center, Università degli Studi di Milano, Milano, Italy.
7. Department of Biotechnology, Jožef Stefan Institute, Ljubljana, Slovenia.
8. Biomedical Research Institute BRIS, Ljubljana, Slovenia.
9. Department of Molecular Genetics, Institute of Pathology, Faculty of Medicine, University of Ljubljana, SI-1000 Ljubljana, Slovenia.
10. Institute of Biochemistry, Faculty of Medicine, University of Ljubljana, SI-1000 Ljubljana, Slovenia.
11. Ljubljana ALS Centre, Institute of Clinical Neurophysiology, University Medical Centre Ljubljana, SI-1000 Ljubljana, Slovenia.
12. Institut du Cerveau et de la Moelle épinière, Inserm U1127, CNRS UMR 7225, Sorbonne Universités, UPMC Univ Paris 06 UMRS1127, Paris, France.
13. Centre de Référence Maladies Rares SLA Ile de France, Département de Neurologie, Hôpital de la Pitié-Salpêtrière, Paris, France.
14. GRC-UPMC SLA et maladies du Motoneurone, France.
15. Ramsay Generale de Santé, Hopital Peupliers, Paris, France.
16. Réseau SLA Ile de France.
17. Instituto de Fisiologia, Instituto de Medicina Molecular,Faculdade de Medicina, Universidade de Lisboa, Lisbon, Portugal
18. Department of Neurosciences, Hospital de Santa Maria-CHLN, Lisbon, Portugal.
19. ALS Unit, Hospital San Rafael, Madrid, Spain
20. Neurology Department, Hospital de la Santa Creu i Sant Pau de Barcelona, Autonomous University of Barcelona, Barcelona, Spain.
21. Centro de Investigación en red en Enfermedades Raras (CIBERER), Spain.
22. Department Neurology, Emory University School of Medicine, Atlanta, GA, USA.
23. Emory ALS Center, Emory University School of Medicine, Atlanta, GA, USA.
24. Euan MacDonald Centre for Motor Neurone Disease Research, Edinburgh, UK.
25. Centre for Neuroregeneration and Medical Research Council Centre for Regenerative Medicine, University of Edinburgh, Edinburgh, UK.
26. School of Medicine, Dentistry and Biomedical Sciences, Queen’s University Belfast, UK.
27. Sheffield Institute for Translational Neuroscience (SITraN), University of Sheffield, Sheffield, UK.
28. Department of Molecular Neuroscience, Institute of Neurology, University College London, UK.
29. Department of Clinical Neuroscience, Institute of Neurology, University College London, UK.
30. Reta Lila Weston Institute, Institute of Neurology, University College London, UK.
31. Department of Neuromuscular Diseases, UCL Queen Square Institute of Neurology.
32. Centre for Neuroscience and Trauma, Blizard Institute, Queen Mary University of London, London, UK.
33. North-East London and Essex Regional Motor Neuron Disease Care Centre, London, UK.
34. Department of Neurology, Hannover Medical School, Hannover, Germany.
35. Department of Neurology, Otto-von-Guericke University Magdeburg, Magdeburg, Germany.
36. Institute of Clinical Neurobiology, University Hospital Wuerzburg, Germany.

37. Charité – Universitätsmedizin, Berlin, Germany.
38. Department of Human Genetics, David Geffen School of Medicine, University of California, Los Angeles, CA, USA.
39. Center for Neurobehavioral Genetics, Semel Institute for Neuroscience and Human Behavior, University of California, Los Angeles, CA, USA.
40. Department of Neurology, David Geffen School of Medicine, University of California, Los Angeles, CA, USA.
41. Department of Neurology, University of California, San Francisco, CA, USA.
42. Center for Neurodegenerative Disease Research, Perelman School of Medicine at the University of Pennsylvania, Philadelphia, PA, USA.
43. Department of Neurology, Perelman School of Medicine at the University of Pennsylvania, PA USA.
44. Koç University, School of Medicine, KUTTAM-NDAL, Istanbul Turkey.
45. Institute of Human Genetics, Helmholtz Zentrum München, Neuherberg, Germany.
46. Centre SLA, CHRU de Tours, Tours, France; UMR 1253, iBrain, Université de Tours, Inserm, Tours, France.
47. APHP, Département de Pharmacologie Clinique, Hôpital de la Pitié-Salpêtrière, France.
48. Université Pierre & Marie Curie, Pharmacologie, Paris VI, Paris, France.
49. BESPIM, CHU-Nîmes, Nîmes, France.
50. Department of Neurology, Ulm University, Ulm, Germany.
51. INSERM U 1127, Hôpital de la Pitié-Salpêtrière, 75013 Paris, France.
52. CNRS UMR 7225, Hôpital de la Pitié-Salpêtrière, 75013 Paris, France.
53. Sorbonne Universités, Université Pierre et Marie Curie Paris 06 UMRS 1127, Hôpital de la Pitié-Salpêtrière, 75013 Paris, France.
54. Institut du Cerveau et de la Moelle épinière, Hôpital de la Pitié-Salpêtrière, 75013 Paris, France.
55. APHP, Département de Génétique, Hôpital de la Pitié-Salpêtrière, 75013 Paris, France.
56. Genethon, CNRS UMR 8587 Evry, France.
57. Department of Medical Genetics, l'Institut du Cerveau et de la Moelle Épinière, Hoptial Salpêtrière, 75013 Paris, France.
58. Department of Neurogenetics, Institute of Neurology, University College London, UK.
59. PopGen Biobank and Institute of Epidemiology, Christian Albrechts-University Kiel, Kiel, Germany.
60. Institute of Clinical Molecular Biology, Kiel University, Kiel, Germany.
61. Department of Genetic Epidemiology in Psychiatry, Central Institute of Mental Health, Faculty of Medicine Mannheim, University of Heidelberg, Germany
62. Institute of Human Genetics, University of Bonn, Bonn, Germany.
63. Department of Genomics, Life and Brain Center, Bonn, Germany.
64. Division of Medical Genetics, University Hospital Basel, University of Basel, Basel, Switzerland.
65. Department of Biomedicine, University of Basel, Basel, Switzerland.
66. Institute of Neuroscience and Medicine INM-1, Research Center Juelich, Juelich, Germany.
67. University of Lille, Inserm, CHU Lille, Institut Pasteur de Lille, U1167 - RID-AGE - Risk Factor and molecular determinants of aging diseases, Labex Distalz, F-59000 Lille, France.
68. Bordeaux University, ISPED, Centre INSERM U1219-Epidemiologie-Biostatistique & CIC-1401, CHU de Bordeaux, Pole de Sante Publique, Bordeaux, France.

69. Department of Internal Medicine, Genetics Laboratory, Erasmus Medical Center Rotterdam, Rotterdam, The Netherlands.
70. Department of Epidemiology, Erasmus Medical Center Rotterdam, Rotterdam, The Netherlands.
71. Department of Epidemiology, Harvard T.H. Chan School of Public Health, Boston, MA, USA.
72. Social, Genetic & Developmental Psychiatry Centre, Institute of Psychiatry, Psychology & Neuroscience, King's College London, London, UK.
73. NIHR Maudsley Biomedical Research Centre, Maudsley Hospital and Institute of Psychiatry, Psychology & Neuroscience, King's College London, London, UK.
74. Amsterdam UMC, Department of Neurology, Location AMC, University of Amsterdam, Neuroscience, Amsterdam
75. Neuromuscular Diseases Unit/ALS Clinic, Kantonsspital St. Gallen, 9007, St. Gallen, Switzerland.
76. Genomic and post-Genomic Center, IRCCS Mondino Foundation, Pavia, Italy.
77. General Neurology, IRCCS Mondino Foundation, Pavia, Italy
78. Neurologic Unit, IRCCS Foundation Ca' Granda Ospedale Maggiore Policlinico, Milan, Italy.
79. Department of Health Sciences, Interdisciplinary Research Center of Autoimmune Diseases, UPO, Università del Piemonte Orientale, Novara, Italy.
80. Department of Neurosciences, University of Padova, Padova, Italy.
81. ALS Centre Department of Neurology Maggiore della Carità University Hospital, Novara.
82. Unit of Genetics of Neurodegenerative and Metabolic Diseases, Fondazione IRCCS Istituto Neurologico 'Carlo Besta', Milano, Italy.
83. "Rita Levi Montalcini" Department of Neuroscience, ALS Centre, University of Torino, Turin, Italy.
84. Azienda Ospedaliera Città della Salute e della Scienza, Torino, Italy.
85. Department of Clinical research in Neurology, University of Bari "A. Moro", at Pia Fondazione "Card. G. Panico", Tricase (LE), Italy.
86. NEMO Clinical Center, Serena Onlus Foundation, Niguarda Ca' Granda Hostipal, Milan, Italy.
87. Medical Genetics Unit, Department of Laboratory Medicine, Niguarda Ca' Granda Hospital, Milan, Italy.
88. Department of Neurology, Institute of Experimental Neurology (INSPE), Division of Neuroscience, San Raffaele Scientific Institute, Milan, Italy.
89. Neurology Unit, Department of Clinical and Experimental Sciences, University of Brescia, Italy.
90. Neurology Unit, Department of Neuroscience and Vision, Spedali Civili Hospital, Brescia, Italy.
91. A list of members and affiliations appears at the end of this Supplementary Note.
92. Centre for Motor Neuron Disease Research, Faculty of Medicine and Health Sciences, Macquarie University, Sydney, New South Wales, Australia.
93. University of Sydney, ANZAC Research Institute, Concord Hospital, Sydney, New South Wales, Australia.
94. Discipline of Pathology, Sydney Medical School, Brain and Mind Centre, The University of Sydney, New South Wales 2050, Australia.
95. Brain and Mind Centre, The University of Sydney, New South Wales 2050, Australia.
96. Hans-Berger Department of Neurology, Jena University Hospital, Jena, Germany.
97. Institute of Human Genetics, Jena University Hospital, Jena, Germany.
98. Institute of Human Genetics, Medical Faculty, RWTH Aachen University, Aachen, Germany

99. Department of Neurology, Brighton and Sussex Medical School Trafford Centre for Biomedical Research, University of Sussex, Falmer, East Sussex, UK.
100. Laboratory of Neurological Diseases, Department of Neuroscience, Istituto di Ricerche Farmacologiche Mario Negri IRCCS, Milano, Italy.
101. Institute of Neurology, University College of London (UCL), London, UK
102. Department of Basic Medical Sciences, Neuroscience and Sense Organs, University of Bari 'Aldo Moro', Bari, Italy.
103. Unit of Neurodegenerative Diseases, Department of Clinical Research in Neurology, University of Bari 'Aldo Moro', at Pia Fondazione Cardinale G. Panico, Tricase, Lecce, Italy.
104. KU Leuven - University of Leuven, Department of Neurosciences
105. VIB, Center for Brain & Disease Research, Laboratory of Neurobiology, Leuven, Belgium.
106. University Hospitals Leuven, Department of Neurology, Leuven, Belgium.
107. Department of Neurology, University of Massachusetts Medical School, Worcester, MA, USA.
108. Academic Unit of Neurology, Trinity College Dublin, Trinity Biomedical Sciences Institute, Dublin, Republic of Ireland.
109. Department of Neurology, Beaumont Hospital, Dublin, Republic of Ireland.
110. Department of Clinical Science, Neurosciences, Umeå University, Sweden.
111. Federation des Centres SLA Tours and Limoges, LITORALS, Tours, France.
112. INSERM U1253, "iBrain", Université François-Rabelais de Tours, Faculté de Médecine 10, Bd Tonnellé, 37032 Tours Cedex 1, France.
113. Department of Translational Neuroscience, UMC Utrecht Brain Center, University Medical Center Utrecht, Utrecht University, Utrecht, The Netherlands.
114. Department of Medical and Molecular Genetics, King's College London, London, UK.

Italian Consortium for the Genetics of ALS (SLAGEN) members

Daniela Calini, Isabella Fogh, Antonia Ratti, Vincenzo Silani, Nicola Ticozzi, Cinzia Tiloca, Barbara Castellotti, Cinzia Gellera, Viviana Pensato, Franco Taroni, Cristina Cereda, Mauro Ceroni, Stella Gagliardi, Giacomo Comi, Stefania Corti, Roberto Del Bo, Lucia Corrado, Sandra D'Alfonso, Letizia Mazzini, Elena Pegoraro, Giorgia Querin, Massimiliano Filosto and Gianni Sorarù

Registro Lombardo Sclerosi Laterale Amyotrofica (SLALOM) group members

Francesca Gerardi, Fabrizio Rinaldi, Maria Sofia Cotelli, Luca Chiveri, Maria Cristina Guaita, Patrizia Perrone, Giancarlo Comi, Carlo Ferrarese, Lucio Tremolizzo, Marialuisa Delodovici, Massimiliano Filosto and Giorgio Bono

Piemonte and Valle d'Aosta Registry for Amyotrophic Lateral Sclerosis (PARALS) group members

Stefania Cammarosano, Antonio Canosa, Dario Cocito, Leonardo Lopiano, Luca Durelli, Bruno Ferrero, Antonio Bertolotto, Alessandro Mauro, Luca Pradotto, Roberto Cantello, Enrica Bersano, Dario Giobbe, Maurizio Gionco, Daniela Leotta, Lucia Appendino, Roberto Cavallo, Enrico Odddenino, Claudio Geda, Fabio Poglio, Paola Santimaria, Umberto Massazza, Antonio Villani, Roberto Conti, Fabrizio Pisano, Mario Palermo, Franco Vergnano, Paolo

Provera, Maria Teresa Penza, Marco Aguggia, Nicoletta Di Vito, Piero Meineri, Ilaria Pastore, Paolo Ghiglione, Danilo Seliak, Nicola Launaro, Giovanni Astegiano and Bottacchi Edo

Sclerosi Laterale Amyotrofica-Puglia (SLAP) registry members

Isabella Laura Simone, Stefano Zoccolella, Michele Zarrelli and Franco Apollo

Neuroprotection and Natural History in Parkinson Plus Syndromes (NNIPPS) Study group members

William Camu, Jean Sebastien Hulot, Francois Viallet, Philippe Couratier, David Maltete, Christine Tranchant, Marie Vidailhet.

References

1. Abdellaoui, A. *et al.* Population structure, migration, and diversifying selection in the Netherlands. *Eur J Hum Genet* **21**, 1277–1285 (2013).

2. Scrucca, L., Fop, M., Murphy, T. B. & Raftery, A. E. mclust 5: Clustering, Classification and Density Estimation Using Gaussian Finite Mixture Models. *R J* **8**, 289–317 (2016).

3. Sawcer, S. *et al.* Genetic risk and a primary role for cell-mediated immune mechanisms in multiple sclerosis. *Nature* **476**, 214–219 (2011).
