# Supplementary material for: Dutch population structure across space, time and GWAS design
Source: Nat Commun. 2020 Sep 11;11:4556. doi: 10.1038/s41467-020-18418-4 (PMC7486932; doi:10.1038/s41467-020-18418-4)
Supplement: Supplementary file 3 — Reporting Summary [file 41467_2020_18418_MOESM3_ESM.pdf]

nature research

Corresponding author(s): Ross P Byrne and Russell L McLaughlin

Last updated by author(s): Jul 24, 2020

Reporting Summary

Nature Research wishes to improve the reproducibility of the work that we publish. This form provides structure for consistency and transparency in reporting. For further information on Nature Research policies, see Authors & Referees and the Editorial Policy Checklist .

Statistics

For all statistical analyses, confirm that the following items are present in the figure legend, table legend, main text, or Methods section.

n/a	Confirmed	
☐	☒	The exact sample size (n) for each experimental group/condition, given as a discrete number and unit of measurement
☒	☐	A statement on whether measurements were taken from distinct samples or whether the same sample was measured repeatedly
☐	☒	The statistical test(s) used AND whether they are one- or two-sided<br>*Only common tests should be described solely by name; describe more complex techniques in the Methods section.*
☐	☒	A description of all covariates tested
☒	☐	A description of any assumptions or corrections, such as tests of normality and adjustment for multiple comparisons
☐	☒	A full description of the statistical parameters including central tendency (e.g. means) or other basic estimates (e.g. regression coefficient) AND variation (e.g. standard deviation) or associated estimates of uncertainty (e.g. confidence intervals)
☐	☒	For null hypothesis testing, the test statistic (e.g. F, t, r) with confidence intervals, effect sizes, degrees of freedom and P value noted<br>*Give P values as exact values whenever suitable.*
☒	☐	For Bayesian analysis, information on the choice of priors and Markov chain Monte Carlo settings
☒	☐	For hierarchical and complex designs, identification of the appropriate level for tests and full reporting of outcomes
☐	☒	Estimates of effect sizes (e.g. Cohen's d, Pearson's r), indicating how they were calculated

Our web collection on statistics for biologists contains articles on many of the points above.

Software and code

Policy information about availability of computer code

Data collection

All genotype data used in analyses were collected as described in their source studies.

Data analysis

For data analysis we used the following publically available software:
Plink v1.9 (QC, Fst calculation, PCA and logistic regression GWAS)
SHAPEIT v2 (Phasing)
Beagle v4.1 (Phasing; IBD calling (RefinedIBD algorithm))
conform-gt utility version 24May16.cee (Pre-phasing checks: https://faculty.washington.edu/browning/conform-gt.html)
Finestructure v2.0.8 (Chromosome painting; Clustering)
Finestructure R tools version date 04/12/2016 (http://www.paintmychromosomes.com/)
ChromoPainter v2 (Chromosome painting)
GLOBETROTTER version date 31/07/2016 (Admixture timing and ancestry profile estimation)
ADMIXTURE v1.3.0 (Inferring admixture proportions)
IBDSeq r1206 (IBD detection for IBDNe)
IBDNe version 04Sep15 e78 (Estimating effective population size)
EEMs v0.0.0.9 (Estimating effective migration surfaces)
SOURCEFIND v2 (Ancestry profile estimation)
pbwtPaint version 3.0-64c4ffa(Chromosome painting; https://github.com/richarddurbin/pbwt/blob/master/pbwtPaint.c)
R v3.2.3 and v3.5.1 - Packages (mclust v5.4.2; fsmb v0.6.3; dendextend v1.9.0; geosphere 1.5-7; Rtsne v0.15; stats v3.2.3; ggplot2 v3.2.1; ape v5.1; rEEMSplots v 0.0.0.9; sp v1.3-1; rgeos v0.4-2; raster v2.8-19; maptools v0.9-5; RcppEigen v0.3.3.5.0; Matrix v1.2-14; grid v3.5.1; ade4 v1.7-15; Imap v1.32; scales v1.0.0)

For manuscripts utilizing custom algorithms or software that are central to the research but not yet described in published literature, software must be made available to editors/reviewers. We strongly encourage code deposition in a community repository (e.g. GitHub). See the Nature Research guidelines for submitting code & software for further information.

Data

Policy information about availability of data

All manuscripts must include a data availability statement. This statement should provide the following information, where applicable:

- Accession codes, unique identifiers, or web links for publicly available datasets
- A list of figures that have associated raw data
- A description of any restrictions on data availability

Data used in this study are available for academic use through the Project MinE Consortium at https://www.projectmine.com/research/data-sharing/. MS GWAS data used for European reference populations were downloaded from the European Genome-phenome Archive under accession EGAD00000000120.
Data availability subject to any conditions outlined by source studies.

Field-specific reporting

Please select the one below that is the best fit for your research. If you are not sure, read the appropriate sections before making your selection.

☒ Life sciences ☐ Behavioural & social sciences ☐ Ecological, evolutionary & environmental sciences

For a reference copy of the document with all sections, see nature.com/documents/nr-reporting-summary-flat.pdf

Life sciences study design

All studies must disclose on these points even when the disclosure is negative.

Sample size	For the main analysis exploring Dutch population structure and demography (Figures 1-5) samples from a single stratum (sNL3; Post QC N=1626) from a recent ALS GWAS (https://www.ncbi.nlm.nih.gov/pubmed/27455348) were included. We selected this stratum due to the availability of geographical data for the majority of samples, with decent spread across the country enabling us to answer fine scale questions about the population. We note that our sample size is comparable to previous studies exploring local population structure using haplotype sharing methods in a single country. As the analyses are largely exploratory predetermination of sample size was unnecessary.<br><br>For our analysis testing the effectiveness of correcting confounding in a Dutch only GWAS using haplotype sharing metrics (Figure 6A; left panel), we selected the best balanced Dutch strata from the original GWAS (sNL1, sNL3 and sNL4), excluding a cohort that contained a disproportionate number of controls (sNL2; 145 cases 4882 controls) to avoid biasing the GWAS. As the goal here was to determine the relative effectiveness of this correction method compared to fitting SNP PCs as covariates a predetermination of sample size was not needed.<br><br>In our final analyses testing the effectiveness of correcting confounding in an international GWAS using haplotype sharing metrics (Figure 6A; right panel), we included all samples from the source ALS GWAS (N=36052; https://www.ncbi.nlm.nih.gov/pubmed/27455348), hence our sample size was determined by the original GWAS size. As the goal here was to determine the relative effectiveness of this correction method compared to fitting SNP PCs as covariates a predetermination of sample size was not needed.
Data exclusions	For the main analysis exploring Dutch population structure and demography (Figures 1-5) samples from other Dutch strata (sNL1, sNL2 and sNL4) from the source paper (https://www.ncbi.nlm.nih.gov/pubmed/27455348) were excluded to avoid potential genotyping platform biases. As our goal was to detect population structure and signals of demographic change we favoured clean data over sample size. Additionally samples and SNPs failing QC described in methods were excluded.<br><br>As noted the methods the analysis investigating the effectiveness of correcting GWAS confounding in a Dutch only sample using haplotype sharing metrics (Figure 6A; Left panel), samples from stratum sNL2 of the original GWAS were excluded due to case control imbalance.
Replication	Our study did not include a biological replication of individual analyses as it is mainly focused on measuring descriptive population features and statistics using cutting edge techniques to improve our understanding of Dutch population genetics. Notably however we do replicate several findings from previous studies of the Netherlands (e.g population expansion, north-south structure). Additionally, our study demonstrates several examples of validation of results on the same sample with different methods to ensure our results are robust. Population clusters identified with fineSTRUCTURE (Figure 1) are largely reproduced by IBD clustering in our study (Figure 3), suggesting they are real features of the data. The signal of exponential growth in population size and the signal of population crash corresponding to the Black Death in our data are both replicated across nine provinces (Figure 4) suggesting the signal is robust. The admixture event detected by GLOBETROTTER in our whole country analysis replicates across each of our clusters (Table 1). Ancestry profiles identified with ChromoPainter (Figure 2) are mirrored by ADMIXTURE results (Supp Fig 3). Ancestry profiles detected using the NNLS method are near identical when estimated using the SOURCEFIND method. Finally our observation that haplotypic PCs explain more variance in case control status in a single population Dutch GWAS is replicated in a larger international GWAS (Figure 6A).
Randomization	The majority of analyses (e.g. detection of finescale structure, ancestry analysis, migration surface analysis, admixture dating, population size analysis) did not involve experimental groupings and instead were performed in all samples to derive descriptive statistics. As a result we did not need randomisation for these analyses. For analyses involving several groups (e.g. regional population size analysis) these groups were a predefined measure such as province of origin and not an experimental treatment, and hence did not require randomisation. For comparison of adjustment of GWAS statistics using haplotype sharing PCs versus SNP based PCs as covariates the same samples were used for each treatment, hence randomisation was not necessary here.

Blinding	Blinding was not relevant to our study as groupings (population clusters) were defined algorithmically.

Reporting for specific materials, systems and methods

We require information from authors about some types of materials, experimental systems and methods used in many studies. Here, indicate whether each material, system or method listed is relevant to your study. If you are not sure if a list item applies to your research, read the appropriate section before selecting a response.

Materials & experimental systems

n/a	Involved in the study
[x]	[ ] Antibodies
[x]	[ ] Eukaryotic cell lines
[x]	[ ] Palaeontology
[x]	[ ] Animals and other organisms
[ ]	[x] Human research participants
[x]	[ ] Clinical data

Methods

n/a	Involved in the study
[x]	[ ] ChIP-seq
[x]	[ ] Flow cytometry
[x]	[ ] MRI-based neuroimaging

Human research participants

Policy information about studies involving human research participants

Population characteristics	Samples originate from a published study with a full breakdown of relevant population covariates (https://www.ncbi.nlm.nih.gov/pubmed/27455348)
Recruitment	Participants were recruited as described in the source paper for the data (https://www.ncbi.nlm.nih.gov/pubmed/27455348).
Ethics oversight	Sample collection and data sharing were approved by country-specific institutional review boards as detailed in the source study (https://www.ncbi.nlm.nih.gov/pubmed/27455348).

Note that full information on the approval of the study protocol must also be provided in the manuscript.
