# Supplementary material for: Dutch population structure across space, time and GWAS design
Source: Nat Commun. 2020 Sep 11;11:4556. doi: 10.1038/s41467-020-18418-4 (PMC7486932; doi:10.1038/s41467-020-18418-4)
Supplement: Supplementary file 2 — Peer Review File [file 41467_2020_18418_MOESM2_ESM.pdf]

Reviewers' comments:

Reviewer #1 (Remarks to the Author):

This is a well-executed study on the population structure of the Dutch population, replicating findings from previous work and refining our knowledge of the genetic structure of the Dutch population. I think it is a good manuscript overall, but I have some points for improvement below.

- The authors mention the use of rare variants in genetic association studies a number of times as a motivation for their endeavors. The SNPs they analyze however have been filtered to have a MAF > .05, which means they include only *very* common variants. The authors should not create the impression that their findings are generalizable to rarer variants unless they also include rare variants in their analyses.
- The authors say in their introduction "We show that Dutch population structure is much stronger than previously recognised". This is quite a strong claim and I do not understand where this comes from. There have been several studies showing how strong the Dutch population structure is (one even showing patterns that are not detected here using a larger sample size, see next comment), and I do not see how this study shows it to be stronger than recognized in the previous studies they cite?
- The two largest patterns of genetic variation from Abdellaoui et al (2013) have been replicated, namely the north-south gradient and the east-west gradient. Abdellaoui et al (2013) however finds a third patterns, namely a strip going through the middle (see their Figure 1). Abdellaoui et al only find this pattern after minimizing LD patterns, allowing their PCA to pick up smaller patterns of ancestral genetic variation. Could the fact that this third geographic pattern is not detected in the current dataset be related to the larger sample size of Abdellaoui et al (N >4000) or could it be a methodological issue? I think this is worth further exploring or discussing this in their article.
- Related to my previous comment, the text and Figure 1 only discuss the first two PCs. I am curious as to whether the other PCs also show significant geographic clustering?
- The authors say "remarkably, some FST values between Dutch clusters were comparable in magnitude to estimates between European countries". I find this a somewhat surprising finding. Are the authors sure that this is not a statistical artefact, perhaps caused by the different sample sizes or some other differences between the Dutch and the European datasets? Since it's such a strong claim and surprising finding, I would like to see some alternative explanations explored/discussed by the researchers.
- The difference in effective population sizes between the North and the South are in line with the relationship between the North-South gradient in homozygosity discussed in Abdellaoui et al (2013), which is interpreted as a consequence of the serial founder effect that is also visible when comparing Northern Europe with Southern Europe (see their discussion and their Figure 2).
- Figure 4 is very nice :)
- I am very surprised at how much variation their PCs explain in the phenotypic variance (Figure 6). Does this mean that 20% of the variance in ALS is explained by ancestry differences? That seems like a gross overestimation, I would like to hear/read more about where the authors think this high estimate comes from.
- In the discussion, the authors discuss the cultural division between the predominantly Catholic South and the Protestant north. This is a well-written discussion, but misses the role of assortative mating, which is extremely high for religious denomination and is expected to further facilitate the existing genetic differentiation between the North and the South (see also https://www.ncbi.nlm.nih.gov/pubmed/23978897 where this is discussed and analyzed in more detail).
- At the end of the discussion, the authors say: "For example, a recent study of latent structure in the UK Biobank demonstrated that a GWAS for birth location returned significant loci even after correction for 40 single marker PCs, suggesting that residual fine-grained population structure may influence other GWAS from this cohort." A recent article in Nature Human Behavior showed that such a signal is actually likely to be a result of the fact that actual complex trait variation is significantly geographically clustered in the UK, largely due to SES-driven migration, see:

https://www.nature.com/articles/s41562-019-0757-5

Reviewer #2 (Remarks to the Author):

The manuscript by Byrne et al. provides a detailed analysis of ancestry in in a large cohort of Dutch individuals. Specifically, they apply haplotype-based methods (ChromoPainter/FineStructure/Globetrotter) to infer fine-scale population structure within the Netherlands. The authors make several interesting findings, including strong north to south genetic structure, as well as more transient west to east population structure. Lastly, they also evaluate the effect of incorporating principal components (PCs) computed from an haplotype-based matrix as covariates in a GWAS setting. They report that PCs from haplotype-based matrices are better at controlling for inflation than the more commonly used PCs obtained from unlinked SNPs.

Overall, I found this manuscript to be quite exciting, and contains many novel and interesting findings. However, I have a number of concerns about the manuscript that I outline below.

My main concern is the FineStructure and total variation distance (TVD) analyses. The authors mention that they identify a total of 40 genetic clusters (k=40) in the Dutch cohort, and that they decided to use the tree formed at k=16 as it captured the main major regional differences. The authors further state that the genetic clusters at this level were supported by their TVD analysis. Yet, the tree presented in the Supplementary Figure 5 based on the TVD matrix is different from the tree obtained by FineStructure presented in Figure 1 (main text). Given that their main results and discussion are based on the FineStructure dendogram, it would be important for the authors to clarify this apparent inconsistency.

Also, although non-negative least squares (NNLS) function are commonly used to produce clean painting profiles, a recent study by Chacon-Duque et al. (2018) presented a novel implementation termed SOURCEFIND. The authors showed through extensive simulations that SOURCEFIND has greater accuracy than NNLS. I would encourage the authors to reassess their observations using this new model if possible.

Also, one of their findings related to their Fst analysis are the large values found between Dutch clusters that sometime outmeasure those observed between European countries. It seems however, that the Fst values were calculated based on a different dataset (i.e. different set of SNPs). If this is the case, I wonder whether the use of a different dataset with distinct common and rare SNPs could have affected the Fst values. The authors could recomputed the Fst values using the overlapping SNPs in their Dutch cohort and the European samples to make them more comparable.

Regarding the IBD analyses. Although I find the complementary analysis to ChromoPainter using refinedIBD very interesting, I find it a bit convoluting to apply a PCA on the IBD similarity matrix, and then use Gaussian Mixture models to identify clusters. Why not perform a hierarchical clustering on the IBD matrix? What is the advantge of computing a PCA first? Also, the ‘mclust’ package uses different models to fit Gaussian distributions, e.g. assuming equal or unequal variances. How was this analysis performed? Also, when classifying observations, the Gaussian Mixture models provide a probability (support) for each assignment. Was there any threshold use?

Further, on the discussion regarding their IBD analyses the authors state: “We have also introduced a novel use of IBD sharing combined with PCA and Gaussian mixture model-based clustering to characterise changing population structure over time, revealing transient genetic structure layered over strong and stable north-south differentiation in the Netherlands.”

I am not sure whether this combination of an IBD matrix, PCA, and Gaussian mixture models is novel.

For example, a paper by Lawson & Falush (2013) (https://www.annualreviews.org/doi/pdf/10.1146/annurev-genom-082410-101510) includes many analyses and extensive simulations based on the 'mclust' clustering approach. If the analyses presented by the authors here is different, and is indeed novel, the author should clearly specify what the novelty is.

Regarding the GWAS section. I found this analysis quite interesting as I have not seen many studies explore the effect of including PCs produced from a haplotype-based matrix as covariates to mitigate GWAS inflation. The authors showed that the LD score regression intercept is lower using these PCs than the ones obtained from unlinked SNP data. I wonder however, whether the same result would be obtained using another number of PCs as covariates. The authors used the first 20, but I would like to see the results based on other numbers. Also, for this analysis the authors removed related individual using a threshold of π higher than 0.1. This threshold however, is different from the one used for the demographic inference analysis. The authors should mention why they decided to use a different cut-off.

Lastly, on the QC section, it is not clear whether the authors removed SNPs associated to ALS in the Dutch case-control dataset (n=1,626) – the one that was used for their main analysis. For the analysis including the European dataset that contained MS patients, they do mention that they removed the HLA region, as it contains many sites that are associated with the disease. Why do it for one cohort, but not the other? Also, why only remove the HLA region? Does the HLA region contain the only reported associated sites?

Minor comments:

"Recent studies have showcased the power of leveraging shared haplotypes to uncover and characterise previously unrecognised fine-grained genetic structure within populations, yielding novel insights into the demographic composition and history of Britain and Ireland, Finland, Japan, Italy and Spain"

Other recent studies employing haplotype-based methods include one conducted in continental France (https://www.nature.com/articles/s41431-020-0584-1) and another conducted in several Latin American populations (https://www.nature.com/articles/s41467-018-07748-z).

Also, SHAPEIT, ChromoPainter, and Beagle use a genetic map as input. The authors should mention which genetic map was employed.

Regarding the FineStructure analyses. When first estimating the switch rate and mutation rate, it is common to do this only on a subset of chromosomes, and a subset of individuals. Was it done this way, or was this analysis run on the whole autosomes and whole dataset? If the analysis was only run on a subset of individuals, it is important to check whether using a larger sample size do not affect the estimates.

Regarding the IBD analyses. I would prefer if the authors could show in the main text the equation used to estimate the age of the IBD segments. The authors should also present the confidence intervals for these estimated ages.

Reference for "lifespan is 3x~ the generation time" is missing.

I would like to see the GLOBETROTTER curves in the supplementary information.

I would like to see the CV errors curves for the ADMIXTURE analysis in the supplementary information.

Line 106 says, “migration dating” this should be referred to as “admixture dating”.

Dutch population structure across space, time and GWAS design

Response to reviewers

Ross P Byrne, Wouter van Rheenen, Project MinE ALS GWAS Consortium, Leonard H van den Berg, Jan H Veldink, Russell L McLaughlin

Reviewer #1 (Remarks to the Author):

This is a well-executed study on the population structure of the Dutch population, replicating findings from previous work and refining our knowledge of the genetic structure of the Dutch population. I think it is a good manuscript overall, but I have some points for improvement below.

- The authors mention the use of rare variants in genetic association studies a number of times as a motivation for their endeavors. The SNPs they analyze however have been filtered to have a MAF > .05, which means they include only *very* common variants. The authors should not create the impression that their findings are generalizable to rarer variants unless they also include rare variants in their analyses.

> We thank the reviewer for taking the time to read and review our manuscript in such depth.
>
> The SNPs used in our study are indeed common variants; however, haplotypes composed of these common variants cover a broader range of frequencies, including much rarer variation, so we expect the recent population structure we observe using haplotype sharing to at least partially describe patterns of rare variant sharing that may confound association studies. With this in mind, we feel that the two mentions of the issues surrounding rare variation (lines 22 and 43-44) in the introduction are justified as a partial motivation for this work. However, as the reviewer points out, we have not directly tested the hypothesis that haplotype sharing matrices can better correct inflation in rare variants specifically, so we have added the following line to the end of our discussion (lines 297-299):
>
> "Such resources will likely be particularly useful in studies of rare variation, motivating future work exploring the efficacy of such strategies in correcting confounding where rare variation is a factor."
>
> We have also modified the introduction to more clearly state the importance of understanding confounding from rare variation (lines 43-44):
>
> "As well as offering insights into demography and human history, refined population genetic studies are important to identify and adequately control confounding effects in genomic studies of health and disease, especially if spatially structured environmental factors contribute substantially to variance in phenotype, which in particular impacts rare variants."

- The authors say in their introduction "We show that Dutch population structure is much stronger than previously recognised". This is quite a strong claim and I do not understand where this comes from. There have been several studies showing how strong the Dutch population structure is (one even showing patterns that are not detected here using a larger sample size, see next comment), and I do not see how this study shows it to be stronger than recognized in the previous studies they cite?

> We acknowledge that this wording is not perfect; our intention was to express that our study has characterised Dutch population structure at a more granular resolution than previous studies thanks to clustering techniques such as fineSTRUCTURE. For example, we have identified subpopulations existing within a short geographic range both between and within provinces (e.g. within the provinces of North Brabant and South Holland). It is true that the population structure itself is not stronger than previously recognised; we have just subdivided it more finely. We have therefore adjusted our wording to reflect our meaning better (line 46):

"We show that Dutch population structure is **more granular** than previously recognised ..."

In response to the additional patterns identified by previous papers, we now present a new supplementary figure (Supplementary Figure 1), replicating this pattern and presenting additional geographically clustered PCs

- The two largest patterns of genetic variation from Abdellaoui et al (2013) have been replicated, namely the north-south gradient and the east-west gradient. Abdellaoui et al (2013) however finds a third patterns, namely a strip going through the middle (see their Figure 1). Abdellaoui et al only find this pattern after minimizing LD patterns, allowing their PCA to pick up smaller patterns of ancestral genetic variation. Could the fact that this third geographic pattern is not detected in the current dataset be related to the larger sample size of Abdellaoui et al (N >4000) or could it be a methodological issue? I think this is worth further exploring or discussing this in their article.

We see a similar trend of a central strip running through the middle of the Netherlands on PC3 of our haplotype sharing matrix, which we have included in Supplementary figure 1 as requested in the next comment. To facilitate comparison, the style of this supplementary figure follows Abdellaoui et al figure 1, however we note that as our PCA is of a haplotype sharing matrix rather than single marker PCA, the trend should not be expected to be identical. Additionally, the haplotype sharing matrix shows further geographic trends, including PCs driven primarily by Utrecht (PC4), Limburg vs North Brabant (West) (PC5), Limburg (PC8) and North Holland (PC9) which is statistically supported by Moran's I (Moran's I $P<0.0001$ for each).

- Related to my previous comment, the text and Figure 1 only discuss the first two PCs. I am curious as to whether the other PCs also show significant geographic clustering?

Additional PCs are now included in Supplementary figure 1 and show significant geographic clustering as described in response to the previous comment. We have added a line in the text to point to this figure for those interested in further PCs (lines 69-70):

"Further PCs demonstrated more complex relationships with geography (Supplementary figure 1)."

- The authors say "remarkably, some FST values between Dutch clusters were comparable in magnitude to estimates between European countries". I find this a somewhat surprising finding. Are the authors sure that this is not a statistical artefact, perhaps caused by the different sample sizes or some other differences between the Dutch and the European datasets? Since it's such a strong claim and surprising finding, I would like to see some alternative explanations explored/discussed by the researchers.

In response to this and a comment by reviewer 2 we have rerun this analysis using only overlapping SNP sets between the Dutch and European datasets (Supplementary table 1), as well as a fixed downsample of individuals (N=29 per group), which correlated strongly (r^2=0.99) with the full analysis. We ran the latter analysis as a "sanity check" and have not presented these results in the manuscript. Results remain consistent, suggesting that this is not a statistical artefact resulting from SNP set or sample size. To clarify, our claim is that some particularly differentiated Dutch cluster pairs, typically those separated by the migrational boundary we identify in figure 5 (eg LIM and NHFG) have a pairwise F_{ST} on the same magnitude of European countries, ie on the order of 10^{-3} (see the newly added supplementary table 1). The mean F_{ST} value across all Dutch cluster pairs is lower than across all European countries, as expected.

Our results illustrate the effectiveness of fineSTRUCTURE at identifying specific Dutch subpopulations that are homogenous enough to be more highly differentiated from one another than some whole (non-subdivided) European populations. Indeed, our findings are consistent with the current literature regarding F_{ST} between fineSTRUCTURE clusters from a single country, eg recent work in Italy which showed that F_{ST} estimates between Italian fineSTRUCTURE clusters are comparable to those between wider European groups (Raveane et al. 2019).

To clarify that our claim here refers to highly differentiated fineSTRUCTURE clusters we have modified the text to state (line 60):

"remarkably, some F_{ST} values between **particularly differentiated** Dutch clusters were comparable in magnitude to estimates between European countries"

We have also updated the methods and Figure 1 to reflect the use of this SNP set.

- The difference in effective population sizes between the North and the South are in line with the relationship between the North-South gradient in homozygosity discussed in Abdellaoui et al (2013), which is interpreted as a consequence of the serial founder effect that is also visible when comparing Northern Europe with Southern Europe (see their discussion and their Figure 2).

The current discussion acknowledges the consistency between the differences in effective population sizes in the North and South and the North-South gradient in homozygosity discussed in Abdellaoui et al. 2013 (lines 225-228):

"Historical N_e follows the same approximate trajectory for both populations but is consistently lower for the northern cluster, corroborating previous observations of increased homozygosity in northern Dutch populations[1] and consistent with a model of northerners representing a founder isolate from southerners (although a more complex demographic model may better explain these observations)[1,2]."

Here, reference 1 is Abdellaoui et al 2013, and Reference 2 is GONL where a similar model was later discussed.

- Figure 4 is very nice :)

Thanks!

- I am very surprised at how much variation their PCs explain in the phenotypic variance (Figure 6). Does this mean that 20% of the variance in ALS is explained by ancestry differences? That seems like a gross overestimation, I would like to hear/read more about where the authors think this high estimate comes from.

In this analysis, Nagelkerke's R^2 is describing a composite estimate of population stratification (non-uniform balance of cases and controls across [sub]populations) and disease heterogeneity across ancestries. This is estimated by fitting a logistic regression of case-control status versus PCs for SNP-PCA and ChromoPainter-PCA, with Nagelkerke's R^2 representing phenotypic variance explained by PCs. Under the assumption of no stratification and no ancestral heterogeneity, this should be zero, with deviations capturing the extent to which stratification and ancestral heterogeneity can be estimated by SNP- or ChromoPainter-PCA.

Our analysis shows that standard SNP-PCA explains ~10% of the phenotypic variance, while ChromoPainter-PCA explains ~20%, indicating that ChromoPainter-PCA is more effective at describing population stratification and/or ancestral heterogeneity. However, there is little evidence to support causal heterogeneity across ancestries in ALS to date; conversely, population structure has consistently been a pervasive source of inflation so we interpret our results as support for ChromoPainter-PCA as a better control for population stratification than SNP-PCA. Additional support is provided by LD score regression, which shows an intercept closer to one upon correction with ChromoPainter-PCA, suggesting that fitting ChromoPainter-PCs to the model reduces inflation caused by confounding.

We have updated the manuscript to include the possibility that disease-ancestry interaction may partially explain our findings (line 280), however we stress that this would have similar implications for the use of standard PCA as a correction method.

- In the discussion, the authors discuss the cultural division between the predominantly Catholic South and the Protestant north. This is a well-written discussion, but misses the role of assortative mating, which is extremely high for religious denomination and is expected to further facilitate the existing genetic differentiation between the North and the South (see also https://www.ncbi.nlm.nih.gov/pubmed/23978897 where this is discussed and analyzed in more detail).

We thank the reviewer for their suggestion. We have included a reference in the discussion to the possible contribution of assortative mating, including this paper (line 245):

"...our data support a model in which the Protestant Reformation of the 16th and 17th centuries exploited pre-existing demographic subdivisions, leading to correlation between distinct cultural affinities and clusters of genetic similarity which has potentially been further strengthened by assortative mating among religious groups[32]."

- At the end of the discussion, the authors say: "For example, a recent study of latent structure in the UK Biobank demonstrated that a GWAS for birth location returned significant loci even after correction for 40 single marker PCs, suggesting that residual fine-grained population structure may influence other GWAS from this cohort." A recent article in Nature Human Behavior showed that such a signal is actually likely to be a result of the fact that actual complex trait variation is significantly geographically clustered in the UK, largely due to SES-driven migration, see: https://www.nature.com/articles/s41562-019-0757-5

We thank the reviewer for this suggestion also. We acknowledge the need to discuss the role of SES-driven migration as a partial cause for the significant loci in the GWAS of birth location in the UK Biobank. We have added a line to the discussion to reference this which should highlight the unanswered challenge of disentangling the effects of fine-grained population structure and stratified lurking variables like migration due to SES (line 294):

"…suggesting that residual fine-grained population structure may influence other GWAS from this cohort (although others suggest a role for socioeconomically-driven migration in this phenomenon[34])."

Reviewer #2 (Remarks to the Author):

The manuscript by Byrne et al. provides a detailed analysis of ancestry in in a large cohort of Dutch individuals. Specifically, they apply haplotype-based methods (ChromoPainter/FineStructure/Globetrotter) to infer fine-scale population structure within the Netherlands. The authors make several interesting findings, including strong north to south genetic structure, as well as more transient west to east population structure. Lastly, they also evaluate the effect of incorporating principal components (PCs) computed from an haplotype-based matrix as covariates in a GWAS setting. They report that PCs from haplotype-based matrices are better at controlling for inflation than the more commonly used PCs obtained from unlinked SNPs.

Overall, I found this manuscript to be quite exciting, and contains many novel and interesting findings. However, I have a number of concerns about the manuscript that I outline below.

My main concern is the FineStructure and total variation distance (TVD) analyses. The authors mention that they identify a total of 40 genetic clusters (k=40) in the Dutch cohort, and that they decided to use the tree formed at k=16 as it captured the main major regional differences. The authors further state that the genetic clusters at this level were supported by their TVD analysis. Yet, the tree presented in the Supplementary Figure 5 based on the TVD matrix is different from the tree obtained by FineStructure presented in Figure 1 (main text). Given that their main results and discussion are based on the FineStructure dendogram, it would be important for the authors to clarify this apparent inconsistency.

We thank the reviewer for their careful review of our manuscript.

As this is the reviewer's main concern we have divided our response into several points to fully address the issue. But to summarise the main point (point 2), the TVD and fineSTRUCTURE trees are expected to differ in topology as outlined in the paper proposing the TVD tree (Kerminen et al. 2017). This is mainly because the fineSTRUCTURE tree can have a dependency on the size of the clusters being considered, favouring the merging of small clusters, while the TVD tree should be largely independent of cluster size as it considers the distance between clusters in terms of mean sharing. However the TVD tree can only compare predefined clusters and is thus dependent on the clustering from fineSTRUCTURE, meaning it is very much a secondary analysis to highlight potential biases in the FS tree topology. We have thus presented both in our manuscript to give a more complete picture of the relationship between clusters. We do not believe the differences in these trees impact the main results or discussion of our manuscript as we do not focus on FS tree topology in the results or discussion, but instead focus primarily on i) the cluster assignments given by step one of fineSTRUCTURE (preceding tree formation); ii) their relationships via complementary methods such as PCA of the ChromPainter matrix, which should both be independent of cluster size. In total the fineSTRUCTURE tree is mentioned once in the results and discussion of our manuscript and we do not derive any major findings from its topology. We have added clarification in the figure legend of the TVD tree (Supplementary figure 7) and mentions of it in the main text.

<u>1) TVD analysis of cluster robustness is not related to TVD tree topology</u>

Firstly we would like to clarify that the TVD analysis which supports genetic clustering at k=16 is the permutation test mentioned on lines 342-347 (in Methods), and is not the TVD tree analysis (discussed below). This test, developed by Leslie et al. (Leslie et al. 2015), compares the TVD (metric of distance) between each pair of fineSTRUCTURE clusters to the TVD of that pair of clusters with their samples randomly permuted across two identically sized clusters. Where the TVD of the fineSTRUCTURE clustering exceeds that of all random permutations, the clusters are said to be well split (i.e. there is a non-random difference between them). When we state that TVD analysis supports our clustering, we mean that for all 1,000 permutations per pair of clusters, our fineSTRUCTURE cluster pairs had a greater TVD than the permuted pairs suggesting they are well defined.

We acknowledge that at submission the TVD tree analysis directly followed this analysis in the methods section with a linking word "Finally" giving the impression that it is part of the cluster robustness analysis, however this was more to indicate that this was the last thing we did with TVD. As such we have reworded this section of the methods to highlight that it is simply an additional method relating to TVD.

<u>2) TVD tree topology is expected to differ from FS tree topology</u>

In order to discuss the differences between the topologies of the fineSTRUCTURE tree and the TVD tree it is important to state two principles of the TVD tree construction: i) it is constructed from predefined clusters (ie it is not a clustering method) and in this case takes the clusters defined by fineSTRUCTURE as its input, and hence is a secondary rather than a primary method; ii) it compares the average sharing between individuals from these predefined clusters, hence it is independent of sample size of those clusters. In contrast, the fineSTRUCTURE algorithm defines the clusters from a set of all individuals by sampling partitions of the haplotype sharing matrix using an MCMC sampler (step 1), and then iteratively merges pairs of clusters from the final set which give the smallest decrease to the model's probability, forming a tree based on the patterns of these merges (step 2). As such the fineSTRUCTURE tree may be dependent on the sample size of clusters, as merging smaller clusters will have a lower impact on the model posterior probability (ie if clusters A B and C are equally genetically distinct, but cluster C is much smaller than A or B, the merge of either cluster with C will result in fewer within-cluster differences than the merge of A and B). To quote the original FS paper on the caveats of the FS tree (Lawson et al. 2012):

> "*Results may depend significantly on sample size, and so should be treated as an approximate guide to similarity, rather than a full population history.*"

Thus the two trees are expected to produce different results, motivating the inclusion of our TVD tree to provide a sample size-independent summary of cluster relationship for completeness. However it should be also be noted that the TVD tree only captures the mean differences between clusters, and hence it could miss some nuances captured by the fineSTRUCTURE tree if there is substantial variation in haplotype sharing within clusters.

3.) Our conclusions do not depend on the FS tree topology, but instead on the clusters

For the majority of the manuscript we do not discuss the fineSTRUCTURE tree topology to derive our results. The primary motivation for this is the quote in point two warning against overinterpretation of the FS tree. Instead we focus on model free methods for considering the relationship between clusters, such as PCA in our discussion and results, given these should be free from bias by sample size (limitation of the fineSTRUCTURE tree) while not ignoring within-cluster variation (limitation of the TVD tree). As such we do not believe the differences in these trees majorly impacts our findings.

Also, although non-negative least squares (NNLS) function are commonly used to produce clean painting profiles, a recent study by Chacon-Duque et al. (2018) presented a novel implementation termed SOURCEFIND. The authors showed through extensive simulations that SOURCEFIND has greater accuracy than NNLS. I would encourage the authors to reassess their observations using this new model if possible.

We have reassessed our observations using the SOURCEFIND model as requested; results are plotted in Supplementary figure 8 and correlation between the NNLS and SOURCEFIND methods via linear regression is reported in the supplementary figure legend. The correlation between the two methods is high for the major contributing sources in spite of the fact that NNLS discards values below 0.05 from a given source ($r^2_{DE} = 0.92$; $r^2_{BE} = 0.97$; $r^2_{DK} = 0.71$) and the general trends of ancestry gradients are equivalent, meaning our main findings will not change regardless of which method we present. We decided to report the NNLS results in the main figure/text in our resubmission for the following two reasons 1) NNLS is currently more widely reported, meaning our results will be more readily comparable with the current literature; 2) The NNLS method produces the same result every time it is run making it more readily reproducible, while the SOURCEFIND method has a small but non zero level of stochasticity across runs due to the use of an MCMC sampler.

Also, one of their findings related to their Fst analysis are the large values found between Dutch clusters that sometime outmeasure those observed between European countries. It seems however, that the Fst values were calculated based on a different dataset (i.e. different set of SNPs). If this is the case, I wonder whether the use of a different dataset with distinct common and rare SNPs could have affected the Fst values. The authors could recomputed the Fst values using the overlapping SNPs in their Dutch cohort and the European samples to make them more comparable.

As requested we have recomputed F_{ST} values between Dutch and European groups using the overlapping SNP set between the two datasets. We have updated Figure 1 with values obtained from this analysis and report F_{ST} between Dutch clusters and European countries in Supplementary table 1 (updated). Our observation that certain highly differentiated Dutch clusters show large F_{ST} values that outmeasure those between some European countries remains consistent (Eg F_{ST}[LIM vs NHFG] = 0.00105 while F_{ST}[France vs Germany] = 0.00068, among other examples). We note that the highest F_{ST} between Dutch clusters is typically for those on opposite sides of the migrational barrier from Figure 5. Additionally we note that this trend holds (r^2=0.99) when clusters and country groups are downsampled to the size of the smallest cluster (n=29), suggesting that differences in sample sizes are not driving this result.

Regarding the IBD analyses. Although I find the complementary analysis to ChromoPainter using refinedIBD very interesting, I find it a bit convoluting to apply a PCA on the IBD similarity matrix, and then use Gaussian Mixture models to identify clusters. Why not perform a hierarchical clustering on the IBD matrix? What is the advantge of computing a PCA first? Also, the 'mclust' package uses different models to fit Gaussian distributions, e.g. assuming

equal or unequal variances. How was this analysis performed? Also, when classifying observations, the Gaussian Mixture models provide a probability (support) for each assignment. Was there any threshold use?

To clarify our approach: mclust and PCA are each applied to the IBD similarity matrix separately (ie mclust is not run on the PCs of the IBD matrix, but the matrix itself). The purpose of running PCA on the IBD similarity matrix is largely to visualise the major trends in IBD sharing across the dataset in a model-free manner. In combination with IBD clustering from mclust, PCA enables us to observe the major trends in IBD sharing both between individuals, and between clusters, easing interpretation of both the relationships between the clusters and the spread of samples within the clusters. The use of analogous methods allows more direct comparison with the analysis of the Chromopainter matrix in figure 1.

Motivation for choice of Mclust

We chose mclust as the multivariate normal likelihood model it uses is noted to be quite similar to that used by fineSTRUCTURE (Lawson and Falush 2012). Additionally it has been shown to perform better than hierarchical clustering methods such as UPGMA in simulations (Lawson and Falush 2012). It also has clear criteria for choosing a number of clusters using the model selection criteria as addressed below, compared to hierarchical clustering, which requires choice of tree cut based on visual inspection or some heuristic index of cluster fit such as the Calinski-Harabasz index (Caliński and Harabasz 1974), which requires more human input to assess.

mclust model selection

As the reviewer points out, mclust can fit several different models, which in the multivariate setting can have equal or unequal shape and volume of covariance across groups (Scrucca et al. 2016). Additionally the method must select the optimum number of groups. For our analysis we fit all model types using up to 9 components selecting the optimal model based on the Bayesian information criterion (BIC) as is the default setting for the software. Fitting a range of models in this way is useful as we do not require strongly predefined assumptions about the size, shape or number of clusters in the data.

Certainty thresholds

The reviewer points out that GMMs provide a measure of cluster assignment confidence. In the presented analysis we have not set a probability threshold for assignment of individuals to clusters, instead selecting the cluster with the highest probability of assignment for each individual as we are looking for general population trends in sharing, rather than focusing on any single individual's exact cluster assignment. However we note that the mean assignment probability is > 0.995 for all analyses in figure 3, suggesting most points are confidently assigned. For example in the total IBD sharing analysis only two individuals have lower probability of assignment than 0.99. While we could remove these individuals from the analysis we feel that their inclusion does not substantially affect the results of the analysis or its goal – to verify the structure identified using ChromoPainting with IBD sharing and to explore changes in population structure at different time depths.

Alternative method: hclust

While hierarchical clustering may be appealing in its simplicity, it is limited in its application to our question by its output, which is a dendrogram object rather than final cluster assignments, leaving a non-trivial question of how to cut the tree. Mclust is preferable in this setting as we can choose the number of clusters by comparing model fit with the BIC as described above.

To validate our results and address any concerns the reviewer may have, we reran our analysis for figure 3 using hclust, choosing a tree cut based on the Calinski-Harabasz criterion as in previous work identifying population structure using the hclust method (Lawson and Falush 2012). We found similar results to our mclust approach (Review figure 1), with persistent north-south structure across bins and more transient east-west structure in

the 3-5cM bin. We note that the general trends in our discussion are preserved, suggesting the two clustering methods perform similarly and the trends are not an artefact of our model choice. However we have decided to continue to use mclust in our main text for the reasons listed above. Additionally running hclust requires more human evaluation when selecting a suitable tree cut even when applying the Calinski-Harabasz criteria (requires selection of the optimal value of the index by visual evaluation) hence it is not practical for the sliding window approach we used in our extended analysis which estimates clustering for ~150 different cM windows (bioinf.gen.tcd.ie/ctg/nlibd).

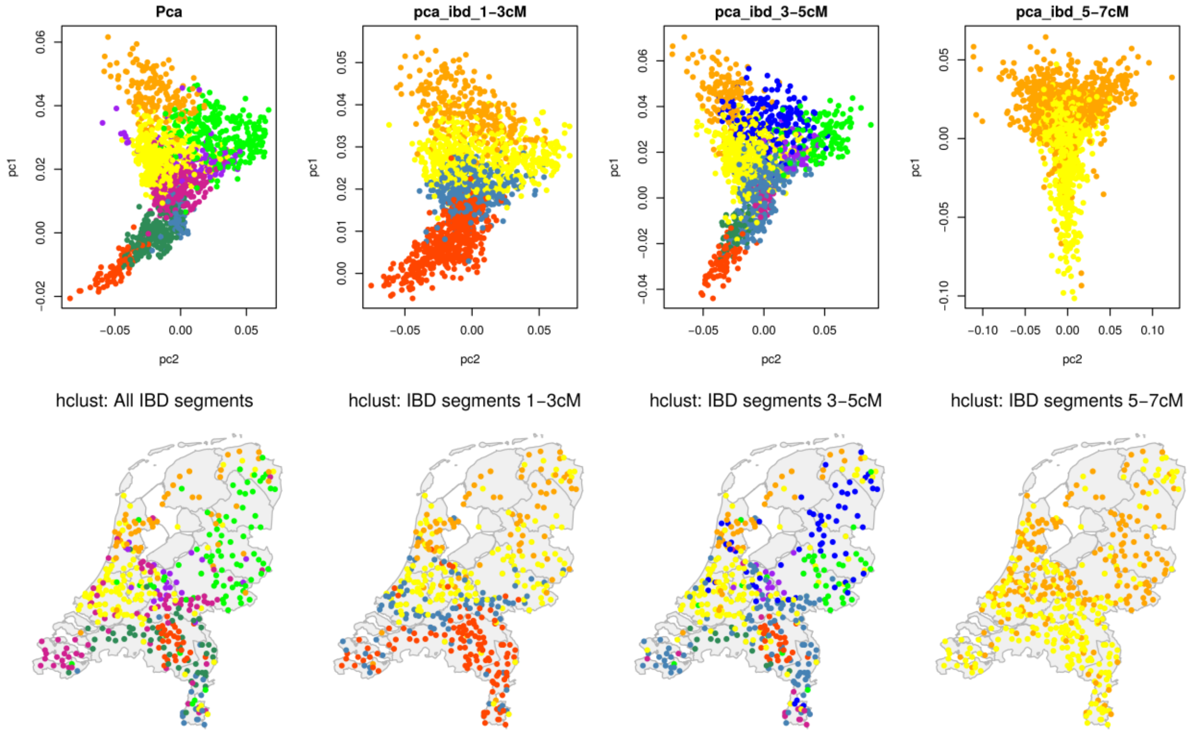

Review figure 1 Clustering analysis from figure 3 on the refinedIBD matrix carried out with hclust (method="ward.D") in place of mclust. Tree cut decided by the maximum Calinski-Harabasz index in the range of k=2-20. Clustering shows comparable structure to mclust.

Further, on the discussion regarding their IBD analyses the authors state: "We have also introduced a novel use of IBD sharing combined with PCA and Gaussian mixture model-based clustering to characterise changing population structure over time, revealing transient genetic structure layered over strong and stable north-south differentiation in the Netherlands."

I am not sure whether this combination of an IBD matrix, PCA, and Gaussian mixture models is novel. For example, a paper by Lawson & Falush (2013) (https://www.annualreviews.org/doi/pdf/10.1146/annurev-genom-082410-101510) includes many analyses and extensive simulations based on the 'mclust' clustering approach. If the analyses presented by the authors here is different, and is indeed novel, the author should clearly specify what the novelty is.

The specific novelty of our method is application of clustering to different IBD segment length bins which enables us to bring an approximate time axis into the analysis based on the expected age of the IBD segments considered. This enables us to look at temporal changes in population structure. We have clarified this in the text (line 180):

"We have also introduced a novel use of **length-binned** IBD sharing combined with PCA and Gaussian mixture model-based clustering to characterise changing population structure over time... "

Regarding the GWAS section. I found this analysis quite interesting as I have not seen many studies explore the effect of including PCs produced from a haplotype-based matrix as covariates to mitigate GWAS inflation. The authors showed that the LD score regression intercept is lower using these PCs than the ones obtained from unlinked SNP data. I wonder however, whether the same result would be obtained using another number of PCs as covariates. The authors used the first 20, but I would like to see the results based on other numbers. Also, for this analysis the authors removed related individual using a threshold of π higher than 0.1. This threshold however, is different from the one used for the demographic inference analysis. The authors should mention why they decided to use a different cut-off.

Additional PCs

We appreciate the reviewer's interest in our analysis of GWAS confounding. We have provided Supplementary figure 12, which displays LD score regression intercepts for analyses fitting 10, 20, 30 and 40 PCs (SNP and haplotypic) as covariates. When only 10 PCs were fitted as covariates the LDSC intercept produced is significantly lower using haplotypic PCs than using SNP PCs. The difference becomes more stark when fitting 20+ PCs. As successive PCs beyond 20 are added we see more minor improvements in the LD score intercept, compared to the jump between fitting 10 and 20 PCs. We expect that this reflects the trend in phenotypic variance explained by successive PCs in figure 6, which in part motivated our initial choice of 20 PCs (The rate of increase in variance explained slows at around this many PCs potentially signalling shrinking returns from fitting additional PCs.)

Relatedness filters

In relation to the individual filtering threshold we originally selected π greater than 0.1 for this analysis as it was used in the source GWAS on the dataset (van Rheenen et al. 2016) and should have approximately removed up to third degree relatives, while enabling us to retain most individuals. However for consistency across the analyses and to reduce biases potentially introduced by cryptically related individuals, we have rerun this analysis using the 0.075 threshold as requested, which removed 230 individuals. Additionally while performing relatedness QC we identified a group of 67 individuals who formed an outlying cluster on PBWT-paint PC1, and had abnormally high chunk sharing between them, who we decided to remove in the reanalysis in case they were cryptically related. The removal of these individuals did not change any results or conclusions. We have updated our methods and figure 6 to reflect these exclusions.

Lastly, on the QC section, it is not clear whether the authors removed SNPs associated to ALS in the Dutch case-control dataset (n=1,626) – the one that was used for their main analysis. For the analysis including the European dataset that contained MS patients, they do mention that they removed the HLA region, as it contains many sites that are associated with the disease. Why do it for one cohort, but not the other? Also, why only remove the HLA region? Does the HLA region contain the only reported associated sites?

The removal of the HLA region associated with MS in the European dataset is consistent with previous work using the dataset (Leslie et al. 2015; Gilbert et al. 2017; Byrne et al. 2018) and is motivated by the strong long range LD in the locus, coupled with strong association with the MS phenotype which together have the potential to bias haplotype sharing profiles significantly. It is not common practice to remove other regions associated with MS when performing ChromoPainter analysis using this dataset as they are not expected to contribute excessively to the sharing profiles, given that they are not subject to long range LD. As there is no other known MS or ALS locus that has similarly long range LD structure we have not removed other disease-associated loci.

Minor comments:

"Recent studies have showcased the power of leveraging shared haplotypes to uncover and characterise previously unrecognised fine-grained genetic structure within populations, yielding novel insights into the demographic composition and history of Britain and Ireland, Finland, Japan, Italy and Spain"

Other recent studies employing haplotype-based methods include one conducted in continental France (https://www.nature.com/articles/s41431-020-0584-1) and another conducted in several Latin American populations (https://www.nature.com/articles/s41467-018-07748-z).

We have included these references and thank the reviewer for pointing them out. The French paper came out after we submitted our manuscript but is a very relevant piece.

Also, SHAPEIT, ChromoPainter, and Beagle use a genetic map as input. The authors should mention which genetic map was employed.

We used the 1000 Genomes phase 3 build 37 as a reference panel and genetic map for all SHAPEIT and ChromoPainter runs, and the Hapmap phase 2 map for Beagle, refinedIBD and IBDNe (as applied in the source papers for refinedIBD and IBDNe). We have included references to these maps in the methods.

Regarding the FineStructure analyses. When first estimating the switch rate and mutation rate, it is common to do this only on a subset of chromosomes, and a subset of individuals. Was it done this way, or was this analysis run on the whole autosomes and whole dataset? If the analysis was only run on a subset of individuals, it is important to check whether using a larger sample size do not affect the estimates.

In our paper we had three main ChromoPainter analyses and estimated mu and switch rate as follows for each run (differences in method of estimation are for computational tractability):

1) Primary Dutch analysis (1626 individuals; ~370k SNPs)

We used all individuals and all chromosomes when estimating the switch and mutation rate for our fineSTRUCTURE run on the 1626 Dutch individuals for the main analysis as this was computationally tractable (relatively few individuals/SNPs).

2.) European/Dutch analysis (6140 individuals; ~150k SNPs)

For the European/Dutch ChromoPainter analysis used in GLOBETROTTER and the ancestry profile estimation we used all individuals and took the weighted mean of mu and N_e estimates from 4 chromosomes to speed up analysis.

3.) Dutch GWAS dataset (4737 individuals, ~1 million SNPs)

For the ChromoPainter analysis of the Dutch GWAS we used the weighted average of four chromosomes again, this time using 10% of individuals (random sample) for our estimates as the large number of variants and individuals led to significant computational burden at the EM stage. N_e and mu estimates were compared to those from our 1626 sample dataset (a larger sample in the same population) and our European/Dutch run (similar number of samples from same demographic root) which were of the same magnitude suggesting our subsample produced sensible estimates.

We have made this explicit in the methods for each analysis.

Regarding the IBD analyses. I would prefer if the authors could show in the main text the equation used to estimate the age of the IBD segments. The authors should also present the confidence intervals for these estimated ages.

We have added the equation to the methods as requested and described our conversion from expected segment age in generations to years. Here we are reporting the expectation of an Erlang-2 probability distribution for the coalescence time for a segment in a given length interval, rather than an empirical estimate from data, so it is problematic to report a meaningful confidence interval for age is as it is would not indicate the confidence of an estimate, but instead the range of 95% of the probability density for the model. Given that theoretically a

segment has a non zero probability of coalescing at any generation between 1 and n, where n is the number of generations considered, this leads to a wide uninformative interval. Our use of point values for expected IBD segment time depths is intended only to guide the reader in a qualitative, population-level comparison of structure between these different temporal ranges and not as a precise estimate of the probability of an IBD segment's temporal provenance.

To make this caveat explicit to the reader, we note in the caption of Figure 3: "Time depths for IBD segment bins have wide distributions[25]; expected values presented here should be interpreted as a guide only and the changing west-east structure over time does not necessarily reflect (for instance) a precisely-timed admixture event."

Reference for "lifespan is 3x~ the generation time" is missing.

This is used as an argument in the introduction of the original IBDNe paper for expected N_e being 1/3 the census size (derived from Felsenstein 1971), where a population N_e is modelled to be lower than the census due to individuals being past reproductive age. If we assume generation time is ~28 years and average lifespan to be 79 years we get 28/79=0.35 which holds. We have added the Felsenstein and IBDNe references as requested (line 442).

I would like to see the GLOBETROTTER curves in the supplementary information.

We have included GLOBETROTTER curves for each of the 12 GLOBETROTTER analyses in table 1 in Supplementary figure 2. For each analysis we have included example curves representing major and minor mixing sources.

I would like to see the CV errors curves for the ADMIXTURE analysis in the supplementary information.

We have added the CV error curve for the ADMIXTURE analysis (Supplementary figure 9). We have also clarified details of the CV run in the methods for the number of folds run (15 in this cases), along with our rationale for selecting a k where the CV-error is tied for lowest, which is to take the model with fewer components for reasons of parsimony.

Line 106 says, "migration dating" this should be referred to as "admixture dating".

We have updated the text to incorporate this suggestion.

REVIEWERS' COMMENTS:

Reviewer #1 (Remarks to the Author):

The authors have done a good job responding to the reviews. I really like the addition of Supplementary Figure 1 (is there room for it to be an additional main Figure?). I recommend accepting this article for publication.

Reviewer #2 (Remarks to the Author):

This is a re-submission of a paper analyzing the fine-scaled genetic structure across the Netherlands, as well as the impact of using haplotype-based methods (instead of the most commonly used allele frequency-based approaches) to correct for population structure in a GWAS setting. The authors have made an excellent job addressing my previous concerns, and I only have two additional minor comments.

Regarding the mclust analyses, I would encourage the authors to show the plots that show the number of components and the BIC values in order to support the number of clusters they show in their main figure (Figure 3).

Lastly, regarding the use of PCs computed from the haplotype-based matrix, I found it interesting that the drop in the LDscore intercept was markedly stronger in the multi-population GWAS setting, compared to the single-population GWAS setting. Naively, I would have expected for the haplotype-based PCs to show an improvement in correcting for population structure in the single-population GWAS setting, as they would have been capturing fine-population structure potentially undetected by the SNP-based PCs. Similarly, I would have expected the SNP-based PCs to be sufficient to capture the major population structure in the multi-population GWAS setting, and not see a big improvement when using haplotype-based PCs. I would encourage the authors to provide potential reasons for this difference, and/or additional analysis (if needed) to support these claims.

Dutch population structure across space and time and GWAS design

Response to reviewers: Round 2

Ross P Byrne, Wouter van Rheenen, Project MinE ALS GWAS Consortium, Leonard H van den Berg, Jan H Veldink, Russell L McLaughlin

Reviewer #1 (Remarks to the Author):

The authors have done a good job responding to the reviews. I really like the addition of Supplementary Figure 1 (is there room for it to be an additional main Figure?). I recommend accepting this article for publication.

We thank the reviewer for their support of our resubmission and believe their comments so far have improved our paper significantly.

We believe that Supplementary Figure 1 should remain a supplementary figure; while it provides further insight into the relationship between the coancestry matrix and geography, we are unsure if it is crucial enough to the main message of the paper or distinct enough from Figure 1 to justify making it a main figure. We defer to the editor on this matter.

Reviewer #2 (Remarks to the Author):

This is a re-submission of a paper analyzing the fine-scaled genetic structure across the Netherlands, as well as the impact of using haplotype-based methods (instead of the most commonly used allele frequency-based approaches) to correct for population structure in a GWAS setting. The authors have made an excellent job addressing my previous concerns, and I only have two additional minor comments.

Regarding the mclust analyses, I would encourage the authors to show the plots that show the number of components and the BIC values in order to support the number of clusters they show in their main figure (Figure 3).

We thank the reviewer for their continued feedback and useful input into our paper. We have included the requested plot of BIC values versus number of components in supplementary figure 9 to support the choice of number of clusters in Figure 3.

Lastly, regarding the use of PCs computed from the haplotype-based matrix, I found it interesting that the drop in the LDscore intercept was markedly stronger in the multi-population GWAS setting, compared to the single-population GWAS setting. Naively, I would have expected for the haplotype-based PCs to show an improvement in correcting for population structure in the single-population GWAS setting, as they would have been capturing fine-population structure potentially undetected by the SNP-based PCs. Similarly, I would have expected the SNP-based PCs to be sufficient to capture the major population structure in the multi-population GWAS setting, and not see a big improvement when using haplotype-based PCs. I would encourage the authors to provide potential reasons for this difference, and/or additional analysis (if needed) to support these claims.

We thank the reviewer for their comment.

Firstly we want to clarify a potential misconception here regarding the reviewer's expectation that there should be little difference in the LDscore intercepts when using haplotype-based PCs or SNP-based PCs in the multi-population GWAS setting. While the major differences between populations may be well described by SNP PCA in this setting, residual fine-scale population structure undetected by SNP-based PCs is not unexpected both within each individual population cohort and between neighbouring or related population cohorts. Hence stratification of cases and controls on this fine scale best captured by haplotypes **would also be expected to inflate GWAS statistics in a multipopulation setting**. Notably residual confounding has been observed following correction with SNP-based PCs in a number of studies, both in a single and multi-population setting, particularly

where multiple small strata are analysed together, supporting the potential role of residual structure following standard correction (Berg et al. 2019; Haworth et al. 2019; Sohail et al. 2019).

We believe the major driver of the apparent differences in LDscore intercept drop in our two settings (single population and multi population) comes down to the scale of the initial confounding. If we take the SNP-PCA corrected GWAS as the baseline for each setting, the multi-population GWAS has a much higher LDscore intercept than the single population GWAS (~1.1 vs ~1.03), hence there is demonstrably more inflation due to confounding not captured by SNP-PCA in this multi-population setting. There is consequently more room for improvement using the haplotype sharing method in the multi-population setting, which may be misinterpreted as the haplotype-based PCA having a stronger effect in the multi-population GWAS. We caution against comparison of the strength of the differences in this analysis.

Given the sample size differences between the single population GWAS (n=4,737) and the multi-population GWAS (n=35,755), and the differences in their "baseline" confounding, we would advise against over interpreting the "differences" in effectiveness of applying haplotype sharing in the single and multi-population GWAS. Such a comparison was not our aim here, nor are these experiments suitably designed to be compared, particularly given the sample overlap (all Dutch samples from the single population GWAS are contained in the multi-population GWAS). The aim of these analyses was simply to address if haplotype-based PCs lowered inflation caused by confounding in the context of each setting. We believe the take home message is that the haplotype sharing method works better than SNP based PCA in a single population and a multi-population setting as it is suitable for correction of both finescale and broadscale population structure. Inferring differences between the multi-population and single population GWAS is beyond the scope of this analysis and would require an extremely large single population cohort to be useful. We therefore feel that further discussion of this difference is not conducive for this study.

<u>References:</u>

1 Berg JJ, Harpak A, Sinnott-Armstrong N, Joergensen AM, Mostafavi H, Field Y et al. Reduced signal for polygenic adaptation of height in UK Biobank. Elife 2019; 8. doi:10.7554/eLife.39725.

2 Haworth S, Mitchell R, Corbin L, Wade KH, Dudding T, Budu-Aggrey A et al. Apparent latent structure within the UK Biobank sample has implications for epidemiological analysis. Nat Commun 2019; 10: 333.

3 Sohail M, Maier RM, Ganna A, Bloemendal A, Martin AR, Turchin MC et al. Polygenic adaptation on height is overestimated due to uncorrected stratification in genome-wide association studies. Elife 2019; 8. doi:10.7554/eLife.39702.
